# Real-World Functional Gait Recovery Trajectories After Total Hip and Total Knee Arthroplasty via Smartphone Sensors: Observational Longitudinal Study

**DOI:** 10.2196/97657

**Published:** 2026-10-30

**Authors:** Levi S Teitz, Einat Granot-Hershkovitz, Pei-An Lee, On-Yee Lo, Brad Manor, Yuval Y Naveh

**Affiliations:** 1 OneStep (Celloscope Ltd), Nachmani 14, Tel Aviv, 6579424, Israel; 2 Marcus Institute for Aging Research, Hebrew SeniorLife, Boston, MA, United States; 3 Beth Israel Deaconess Medical Center, Boston, MA, United States; 4 Harvard Medical School, Harvard University, Boston, MA, United States

**Keywords:** total hip arthroplasty, total knee arthroplasty, gait analysis, smartphone sensors, remote patient monitoring, functional recovery, digital health, observational study, gait parameters

## Abstract

**Background:**

Recovery after total hip arthroplasty (THA) and total knee arthroplasty (TKA) is typically assessed with infrequent clinic-based evaluations that may not reflect patients’ everyday mobility. Smartphone inertial sensors enable scalable, repeated gait measurement in real-world settings, but the extent to which prompted walking tests reflect daily life walking during recovery is not well defined.

**Objective:**

The objective of this study was to characterize week-by-week recovery trajectories after THA and TKA using high-frequency smartphone sensor data and quantify the difference between prompted in-app walking tests and passive daily life background walking.

**Methods:**

We analyzed longitudinal smartphone-based gait measurements from 735 patients (THA, n=350; TKA, n=385) who underwent unilateral surgery between July 2022 and October 2025. Nine spatiotemporal gait parameters were monitored over the first 15 postoperative weeks across 2 conditions: prompted “in-app” walks and passively captured “background” walks. Recovery trajectories were evaluated using Kaplan-Meier time-to-baseline analyses, linear mixed-effects models, and comparison of late-stage plateau levels (weeks 11‐15) using estimated marginal means of the models.

**Results:**

Across all parameters, THA patients returned to preoperative baseline levels faster than TKA patients, with mean trajectories generally approaching baseline by week 4 for THA versus weeks 6 to 8 for TKA. Linear mixed-effects models showed that surgical procedure type significantly influenced recovery trajectory for all parameters except single support of the unaffected leg. All gait parameters except stance asymmetry and single support of the unaffected leg exhibited persistent differences through the late-recovery plateau (weeks 11‐15). We also compared prompted “in-app” walks with passively captured background walking. Prompted and passive walking showed systematic divergence in both THA and TKA; prompted assessments generally indicated faster or more complete return to baseline than passive daily life walking. Finally, preoperative gait level was strongly associated with postoperative trajectories, with baseline-stratified analyses showing distinct patterns of recovery between groups.

**Conclusions:**

High-frequency smartphone gait assessment quantifies procedure-specific recovery dynamics and demonstrates that prompted tests and passive daily life measurements capture distinct, noninterchangeable functional signals. Parameters from prompted walks plateaued while those from passively captured walks were still changing; prompted testing alone therefore captures only one component of returning function, highlighting the additional information that passive, real-world gait monitoring can provide.

## Introduction

Total hip arthroplasty (THA) and total knee arthroplasty (TKA) are highly successful orthopedic surgeries that restore mobility for millions of people with end-stage arthritis [1,2]. Their volume is substantial and growing; over 3.7 million Americans underwent THA or TKA from 2021 to 2024 [3], and projections indicate that the annual volume for THA and TKA could increase by 176% and 139%, respectively, by 2040, compared to 2019 volumes [4]. This escalating demand establishes total joint arthroplasty (TJA) as a major component of the health care system, making the optimization of postoperative recovery a critical public health priority.

Conventional methods for monitoring patient recovery after TJA have significant limitations and provide an incomplete picture of a patient’s functional status [5]. The standard of care relies on infrequent, in-person clinical visits featuring practitioner-dependent assessments such as the Timed Up and Go test [6]. These appointments offer only brief snapshots of function in a controlled setting and correlate poorly with a patient’s actual daily activities. This episodic model limits a clinician’s insight into the patient’s day-to-day progress and challenges, hindering proactive intervention. Furthermore, the common practice of limiting complication surveillance to the acute recovery phase has been shown to miss a substantial number of adverse events, revealing a systemic gap in long-term patient monitoring [7].

Another key challenge in postarthroplasty care is the discordance between subjective patient-reported outcome measures (PROMs) and objective, performance-based metrics [8]. While PROMs are vital for capturing the patient’s perspective on function, pain, and quality of life, studies show that early improvements in these scores often do not align with objectively measured functional gains [9,10]. Patients frequently report feeling significantly better long before objective measures, such as walk speed or mobility, return to normal levels. This may partly reflect that patients often associate recovery with meaningful pain relief, even when objective mobility remains impaired. However, pain relief is unlikely to be the only explanation, and the broader point remains that perceived recovery and measured physical performance can differ substantially. This issue is compounded by low patient completion rates for long-term PROMs, which can introduce bias into the data [11]. This disconnect highlights the need for granular, individualized, and objective monitoring tools that can characterize function beyond self-assessment.

Previous studies have attempted to establish such recovery benchmarks and expected trajectories [12-14]. However, these efforts are often limited in scope, focusing on a single metric or joint, or on limited follow-up periods with infrequent data collection time points, which fail to capture the continuous, dynamic nature of the recovery process.

The widespread availability of smartphones with built-in inertial measurement units (IMUs) offers a validated and scalable solution to the limitations of traditional monitoring. A growing body of research confirms the reliability of smartphone-based systems for measuring key gait parameters like walk speed, cadence, and asymmetry in real-world environments [15-18]. These systems show strong correlation with gold-standard laboratory equipment. This technology shifts measurement from the clinic to the patient’s daily life, enabling the continuous, high-frequency collection of objective functional data that is more representative of true mobility [19]. Digital health platforms like the OneStep system leverage these capabilities to create objective recovery benchmarks and track individual progress with unprecedented detail.

While remote monitoring provides a wealth of objective data, a critical nuance essential for understanding true recovery is often overlooked: the distinction between a patient’s functional capacity—what they are capable of doing under observation or during a prompted task—and their habitual daily performance—what they actually do in an unobserved, unguarded state [20-23]. This distinction is vital, as the goal of rehabilitation is not merely to enable a patient to perform a task correctly, but to restore normal, noncompensatory movement as an automatic pattern in daily life.

This study presents week-by-week gait trajectories after THA and TKA, measured using a smartphone-based system, to guide more precise postoperative monitoring and rehabilitation. Specifically, we compare the 15-week recovery trajectories of THA and TKA patients across 9 gait parameters and quantify differences between prompted in-app walks, treated as a proxy for functional capacity, and passively captured background walking, treated as a proxy for habitual performance.

## Methods

### Cohort

This study analyzed deidentified data collected from commercial users (N=735) of the OneStep gait monitoring system during routine postoperative care and recovery monitoring between July 2022 and October 2025. Data were not collected specifically for this research but were generated as part of standard app use. Starting from all patients with a recorded THA or TKA, we sequentially applied eligibility criteria based on surgery type, data availability, and preoperative recording requirements.

### Ethical Considerations

This study used secondary, deidentified data collected during routine use of a commercial gait monitoring platform. Users provided consent at onboarding for their deidentified data to be used in aggregate for research purposes. The authors determined that the secondary analysis met the criteria for exemption from institutional review board oversight under the US Federal Policy for the Protection of Human Subjects (Common Rule), 45 CFR §46.104(d)(4)(ii) [24]. For the research analysis, information was recorded in a manner such that the identities of individuals could not readily be ascertained directly or through identifiers linked to the data. The investigators did not contact individuals and did not attempt, and will not attempt, to reidentify them. The study was conducted in accordance with applicable privacy and ethical standards.

### Gait Data Collection

Gait was measured before and after surgery with OneStep’s smartphone gait analysis system (software versions 2.18.0‐4.0.0; Celloscope Ltd). Figure 1 shows the measurement screen of the mobile app. Patients carried their phones in a pants pocket while walking. The sensors of the phone’s IMU record 3-dimensional acceleration and gyroscope data at a sampling frequency of 50 Hz and upload the data to secure servers. No calibration is required. The inertial motion data are split into individual strides, and a supervised convolutional neural network then assigns each stride to a joint (physical activity, device body position) class; strides that are not assigned to the walking class or a pants pocket body position are excluded from the analyses reported here [25]. A proprietary deep learning framework is then used to detect which side the phone was carried on and compute standard spatiotemporal gait measures for each step. This framework was trained using a gold-standard gait measurement system (the C-Mill VR+ instrumented treadmill; Motek) to generate ground truth labels for simultaneous phone measurements from a variety of healthy and unhealthy walkers; these results were then validated against a selection of gold-standard gait measurement methods [15-17]. The software is compatible with almost all modern smartphones, as most smartphones contain the necessary IMU hardware, and is compatible with both iOS and Android operating systems. Although the gait analysis system has not been validated specifically in a post-TJA population, it has been validated in cohorts with a variety of moderate to severe gait abnormalities [16,17].

**Figure 1.**
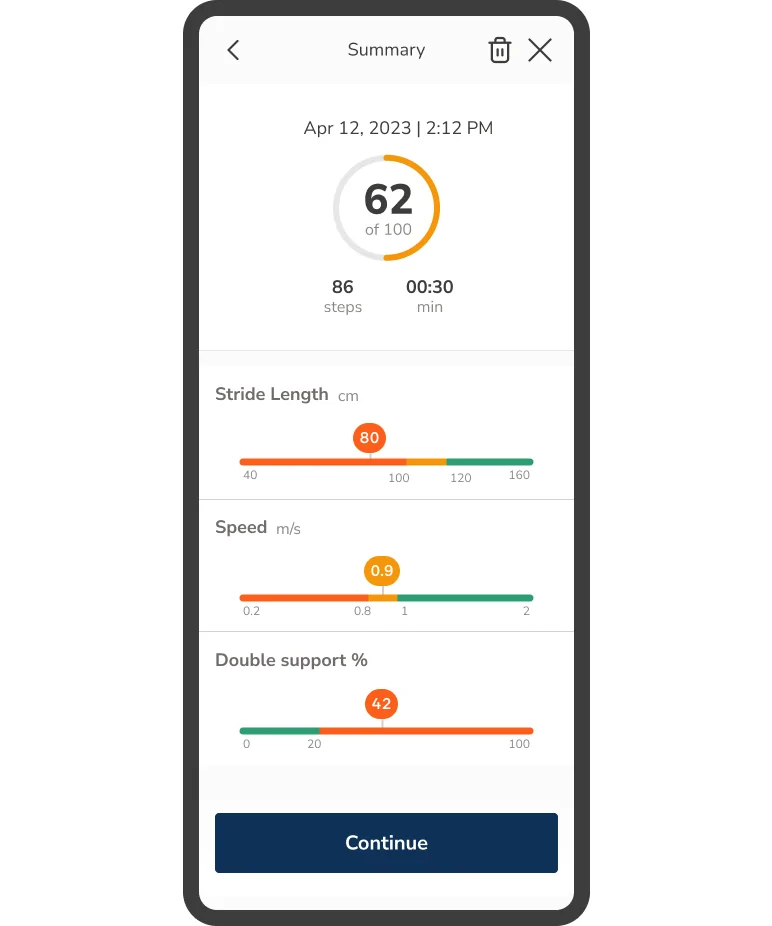
Screenshot of the OneStep mobile app walk analysis screen.

Nine gait parameters were analyzed for this study: cadence, single support percentage of the affected (ie, the leg upon which the operation was performed) and unaffected leg; stance asymmetry; double support percentage; step length of the affected and unaffected leg; stride length; and walk speed. Stance asymmetry is defined as the difference between the stance percentage of the 2 legs; that is, the percent of stride time each leg is in contact with the ground. For this study, it was calculated as the stance percentage of the unaffected leg minus the stance percentage of the affected leg. We used the signed value of stance asymmetry, rather than its absolute value, because the direction of asymmetry is clinically relevant in TJA, where one leg is affected.

### Walk Types

We used 2 kinds of recordings:

1. *In-app walks*. Patients opened the OneStep mobile app, started a recording, placed the phone in a pocket, and walked in a straight line (with 180° turns as necessary) on a flat surface for about 60 seconds.
2. *Background walks*. With permissions enabled, the app ran in the background and automatically recorded motion when it detected pocket-carried walking during daily life.

### In-App Walk Collection

In-app walks were collected before and after surgery when patients chose to initiate a recording; no fixed recording schedule or surgeon-directed reminders were used. Recordings were performed in patients’ own environments. App instructions directed patients to walk on a clear, straight path, to turn around and walk back and forth if the available path was too short, and to walk at a comfortable pace. Data processing and filtering are described above in *Gait Data Collection*.

### Background Walk Collection

Background walks represent a particular challenge for data collection because walks can be taken in a variety of contexts (indoors vs outdoors, with or without shoes, on various terrains, straight-line walking or curved paths, etc) and because the patient can walk with their smartphone in a position other than the pants pocket. In addition, daily walking can occur in short bursts of 5 seconds or less, which have qualitatively different attributes than more extended walking bouts.

To account for these factors, the background gait analysis pipeline performs several steps. First, only walking bouts of 30 seconds or longer are analyzed. Second, any steps taken while not walking in a straight line are filtered out before analysis, and walking bouts with too few straight strides are flagged and filtered out. Straight-line walking was identified from gyroscope-derived heading after the sensor data were oriented to the direction of motion. Third, phone position (pocket vs nonpocket) is detected by a deep learning framework that was trained on examples of motion data from phones placed in the pocket and in a variety of other locations (hand, bag, shirt pocket, etc). Any walk that is detected as not being from the pants pocket is filtered out. Finally, gait measurements may differ between prompted and background conditions, as has been shown previously for this gait analysis system [23]. Therefore, as described below, in-app and background data are normalized separately to their respective presurgery baseline values. By normalizing in-app and background walks separately, lower background values at a given postsurgery week are interpreted relative to the patient’s own presurgery background walks, not relative to their in-app walks. This distinction is important because raw background and in-app values may differ for reasons unrelated to recovery, including phone position, walking environment, and walking path. The analysis therefore asks whether background gait returns to its own baseline more slowly than in-app gait, rather than whether background gait has lower absolute parameter values.

### Preoperative Baseline

Inclusion required at least one in-app gait recording and one background gait recording in the 14 days before surgery; patients without such data were excluded. If multiple recordings were available, their gait measures were averaged to define that patient’s baseline. Baselines were calculated separately for in-app and background walks.

### Aggregation and Normalization

For each patient, postoperative walks were grouped by week since surgery (days 1‐7, 8‐14, etc) and averaged to create weekly values. The patient’s baseline was then subtracted from each weekly value to express change from baseline. To describe population-level recovery, these normalized values were averaged across patients contributing data in that postoperative week. Analyses used available data only (weekly sample sizes varied); no temporal interpolation or imputation was performed.

### Statistical Analysis

To quantify differences between recovery trajectories, 3 methods were used. First, Kaplan-Meier analysis was used to determine whether time to first observed return to preoperative baseline differed between conditions. For parameters where a higher value is considered healthier (cadence, single support, step and stride length, and walk speed), time to return to baseline was considered the first week after surgery during which the parameter value was equal to or higher than the preoperative baseline level; for parameters where a lower value is healthier (double support and stance asymmetry), the first week in which the value was equal to or lower than baseline was used. A sustained crossing was not required because consecutive weekly observations were unavailable for many participants; requiring them would have excluded a large proportion of the cohort. Concretely, the Kaplan-Meier curve for a given parameter shows, for each postoperative week, the fraction of patients who had reached their own presurgery gait level by that week; a curve that rises sooner or reaches a higher fraction indicates faster or more complete return to baseline. A log-rank test (or a multivariate log-rank test, in cases where more than 2 Kaplan-Meier curves were being compared) was used to determine significance.

Second, we used linear mixed-effects models to test whether postoperative recovery trajectories differed between conditions. For each gait parameter, we fitted a model with fixed effects for postoperative week (modeled using a B-spline basis), condition (eg, THA vs TKA; in-app vs background; preoperative baseline level), age, and gender, and included interactions between the spline basis for week and each covariate of interest to test for differences in trajectory shape (time-varying effects) rather than only overall level shifts. A patient-specific random intercept was included to account for repeated measures within individuals. Postoperative week was represented with a B-spline basis of degree 1 and 4 degrees of freedom.

To test the importance of each fixed effect, we performed nested-model likelihood ratio tests by refitting reduced models that excluded one term at a time (condition, age, or gender) and its interactions, holding all remaining fixed effects, the postsurgery week specification, and random effects constant. The likelihood ratio statistic was computed as 2(ℓ_full_ − ℓ_reduced_), where ℓ is the maximized log-likelihood, and *P* values were obtained by comparing this statistic to a chi-square distribution with degrees of freedom equal to the difference in the number of estimated parameters between the full and reduced models.

Third, to compare parameter levels of late-stage plateaus, we computed estimated marginal means (EMMs) from the linear mixed-effects models. We defined the plateau as weeks 11 to 15 postsurgery and, for each condition, obtained the model-predicted mean over this window with age fixed at its sample mean and gender averaged by its observed proportions. Statistical significance of the difference in means between conditions was assessed with a Wald test, from which we report *z* scores and *P* values, along with 95% CIs.

All *P* values for all tests were adjusted using Benjamini-Hochberg false discovery rate correction over all *P* values in the analysis type (Kaplan-Meier, linear mixed-effects models, and EMMs). Statistical and modeling work was performed using Python and the Python packages lifelines, statsmodels, and SciPy; R and the R packages lme4, emmeans, and splines were used for the EMM analysis.

## Results

### Patient Demographics

Data were analyzed from 735 patients who underwent THA (n=350) or TKA (n=385). Baseline characteristics, including age distribution and gender composition used for subgroup analyses, are summarized in Table 1. The 2 cohorts had similar demographics: the THA cohort had a mean age of 69.6 (SD 7.6) years and included 56% (197/350) of women, while the TKA cohort had a mean age of 70.9 (SD 6.9) years and included 59% (227/385) of women. Figure S1 in Multimedia Appendix 1 shows the age distributions of the 2 cohorts. Preoperative baseline parameters differed slightly by gender, age, and procedure (Figure S2 in Multimedia Appendix 1). Figure 2 summarizes cohort assembly and filtering.

**Table 1.** Characteristics of total hip arthroplasty and total knee arthroplasty cohorts.

Characteristic	THA^a^ (n=350)	TKA^b^ (n=385)
Male participants, n (%)	153 (44)	158 (41)
Female participants, n (%)	197 (56)	227 (59)
Age (y), mean (SD)	69.6 (7.6)	70.9 (6.9)
Age of male participants (y), mean (SD)	69.2 (7.8)	70.9 (7.6)
Age of female participants (y), mean (SD)	69.9 (7.4)	70.9 (6.3)
Recorded in-app walks, n	8183	7289
Left-side surgery, n	191	214
Right-side surgery, n	159	171

a THA: total hip arthroplasty.

b TKA: total knee arthroplasty.

**Figure 2.**
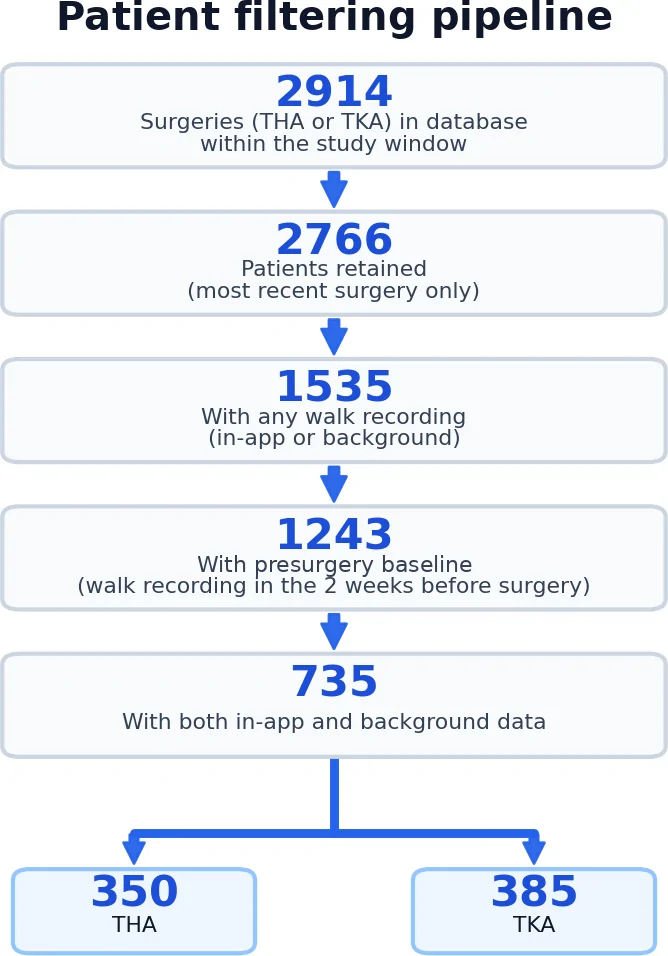
Cohort selection flowchart. Flowchart showing derivation of the analytic cohort from all patients with a recorded THA or TKA. Patients were sequentially filtered according to study eligibility criteria, including procedure type, availability of postoperative gait data, and availability of both in-app and background preoperative recordings required to define baseline. The final cohort was then stratified into THA and TKA groups for analysis. THA: total hip arthroplasty; TKA: total knee arthroplasty.

### Recovery Trajectories After THA Versus TKA

Across all 9 gait parameters, average values relative to the preoperative baseline were lower after TKA than after THA in postoperative week 1. The mean THA trajectory returned to baseline by about week 4, whereas mean TKA trajectories reached baseline later, between weeks 6 to 8 (Figure 3 and Figure S3 in Multimedia Appendix 1). Kaplan-Meier analysis showed significant differences between THA and TKA in time to return to baseline for all parameters except single support of the unaffected leg (Figure S4 in Multimedia Appendix 1). Linear mixed-effects models showed that adding THA versus TKA status significantly improved fit for all gait parameters except single support of the unaffected leg (Figure S5 in Multimedia Appendix 1).

**Figure 3.**
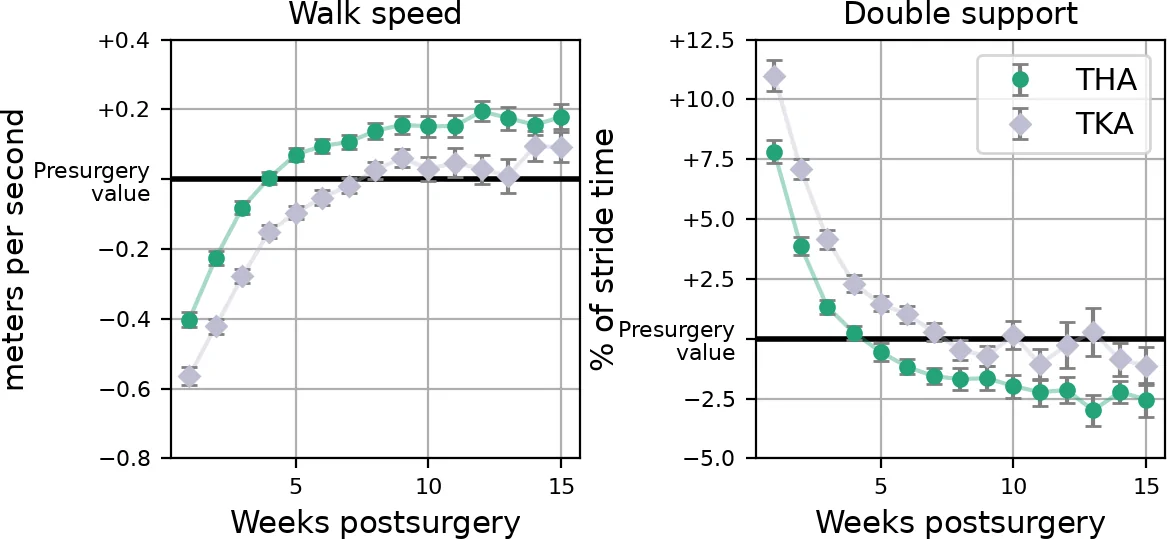
Recovery trajectories of in-app gait parameters after surgery in THA versus TKA. Each data point represents the mean change from all patients’ preoperative baselines for that postoperative week; error bars show SE. Positive values indicate increases relative to baseline. Improvement corresponds to an increase in walk speed and a decrease in double support. For both parameters shown, THA patients returned to their preoperative gait level earlier than TKA patients, consistent with the known differences in procedural invasiveness and rehabilitation trajectory between the 2 surgeries. Weekly counts of patients contributing measurements in each group are given in Table S2 in Multimedia Appendix 1. THA: total hip arthroplasty; TKA: total knee arthroplasty.

After crossing baseline, both procedures showed stable trajectories in weeks 11 to 15, with minimal week-to-week fluctuation. However, on average, THA patients reached a healthier level relative to the preoperative baseline than TKA patients at this plateau stage in all parameters except stance asymmetry and single support of the unaffected leg, and for most parameters, this difference reached an appreciable change in magnitude (Table S1 in Multimedia Appendix 1). For example, the difference in EMM at weeks 11 to 15 for walk speed was 0.14 m/s, exceeding the commonly used 0.1 m/s threshold for a clinically meaningful change in walk speed [26].

In the final weeks, some parameters showed unusual fluctuations or shifts in plateau level. This likely reflects diminishing and variable weekly sample sizes, as not every patient recorded an in-app walk each week and recordings tend to decline over time (Figure 4 and Figure S6 and Table S2 in Multimedia Appendix 1). This incompleteness may similarly affect the other analyses, including the Kaplan-Meier curves, and raises the possibility of survivorship bias if patients who continued contributing data differed systematically from those who did not; we consider these issues further in the *Discussion*.

**Figure 4.**
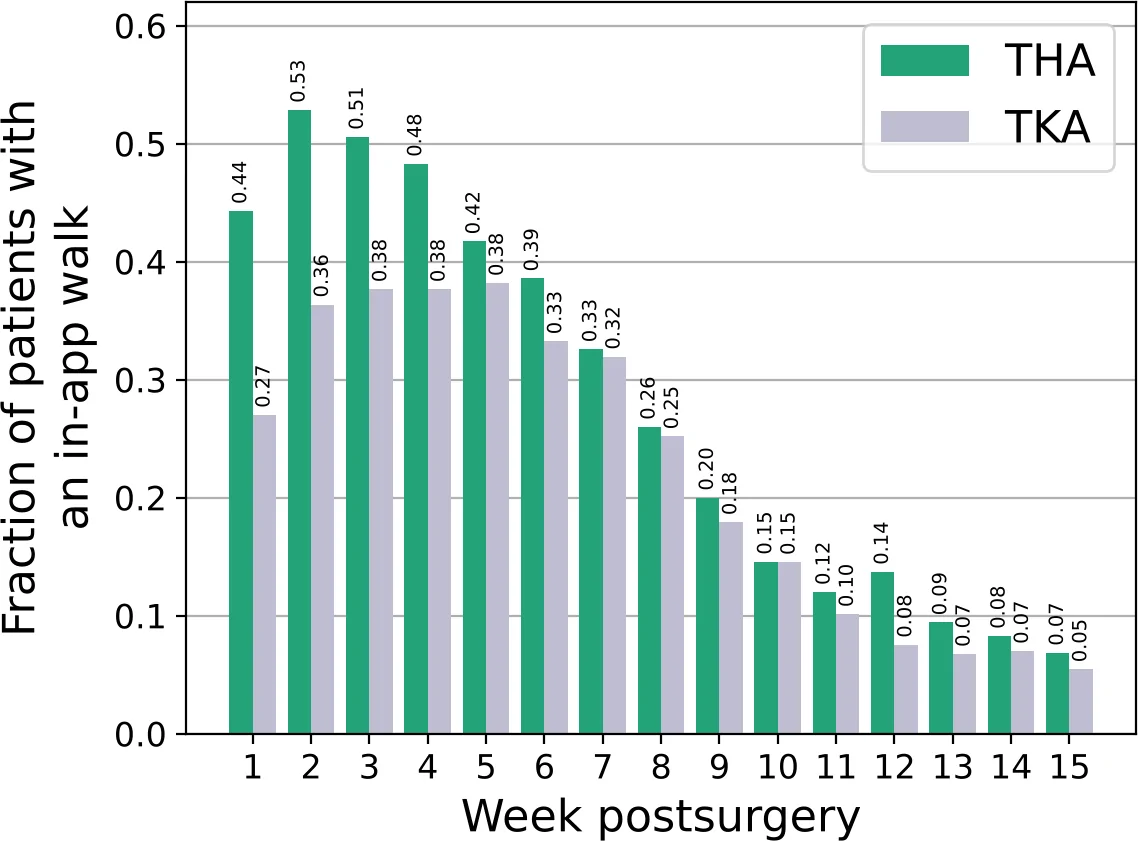
Fraction of patients contributing in-app walks by postoperative week. For each postoperative week, bars show the fraction of patients in each cohort who recorded at least one in-app walk that week, out of all patients in the cohort (THA, n=350; TKA, n=385). Adherence peaks in the early postoperative weeks and falls steadily thereafter in both groups, reaching under 10% by week 15. Absolute weekly patient counts for these groups, as well as for background walks and subgroups, are given in Table S2 in Multimedia Appendix 1. THA: total hip arthroplasty; TKA: total knee arthroplasty.

### Recovery Trajectories of In-App Versus Background Measurements

We next compared recovery trajectories from prompted in-app walks with those from passive background activity. In both THA and TKA, differences in recovery trajectories between these 2 types of walking were observed (Figure 5 and Figure S7 in Multimedia Appendix 1). Kaplan-Meier analysis did not show significant differences in time to return to baseline between in-app and background walking, except for walk speed and step length of the unaffected leg after THA (Figure S8 in Multimedia Appendix 1). However, linear mixed-effects models showed that adding in-app versus background status significantly improved fit for all gait parameters, most at a very high level of significance (Figure S9 in Multimedia Appendix 1). The level of the late-stage plateau differed significantly between in-app and background walking after THA for all parameters except stance asymmetry and single support of the unaffected leg. Effect sizes were generally of a lower magnitude than those of the plateau differences between THA and TKA, although the difference in walk speed was 0.14 m/s, the same as the difference between THA and TKA (Table S1 in Multimedia Appendix 1). The plateau levels after TKA differed significantly between in-app and background walking in cadence, step length of both legs, stride length, and walk speed; however, effect sizes were much lower than even those of in-app versus background walking in THA, including a difference in walk speed of 0.07 m/s (Table S1 in Multimedia Appendix 1).

**Figure 5.**
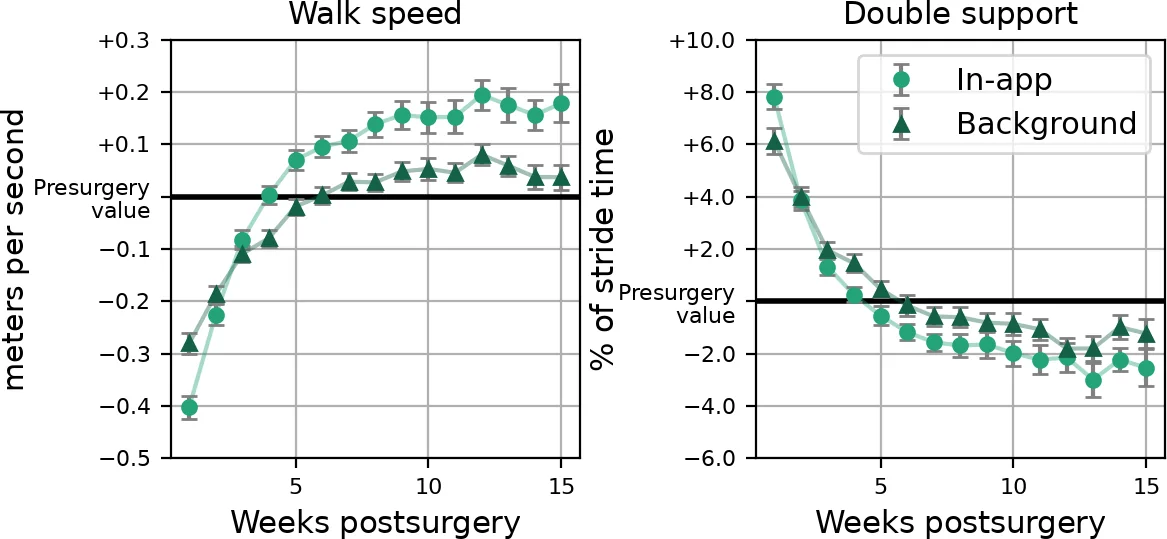
Recovery trajectory of in-app versus background gait parameters after surgery in total hip arthroplasty. Each data point represents the mean change from all patients’ preoperative baselines for that postoperative week; error bars show SE. Positive values indicate increases relative to baseline. Improvement corresponds to an increase in walk speed and a decrease in double support. Background walking returns to baseline more slowly than in-app walking, suggesting that prompted clinical assessments may overestimate a patient’s functional recovery in daily life. Weekly counts of patients contributing measurements in each group are given in Table S2 in Multimedia Appendix 1.

### Recovery Trajectories by Age and Gender

We performed these analyses with populations stratified by gender and age (Figure 6 and Figures S10 and S11 in Multimedia Appendix 1). By gender, trajectories were visually highly similar for THA (male, n=153; female, n=197) and TKA (male, n=158; female, n=227). By age, patterns were likewise highly similar across groups in THA (<65 y: n=86; 65‐75 y: n=173; 75+ y: n=91) and TKA (<65 y: n=76; 65‐75 y: n=200; 75+ y: n=109). Kaplan-Meier analysis showed no difference in time to return to baseline between gender or age groups for any parameter (Figures S12 and S13 in Multimedia Appendix 1). Although adding age or gender to the linear mixed-effects models significantly improved model performance for many parameters (Figures S5 and S9 in Multimedia Appendix 1), the significance of those improvements was generally many orders of magnitude lower than that observed when adding condition (eg, THA vs TKA, in-app vs background).

**Figure 6.**
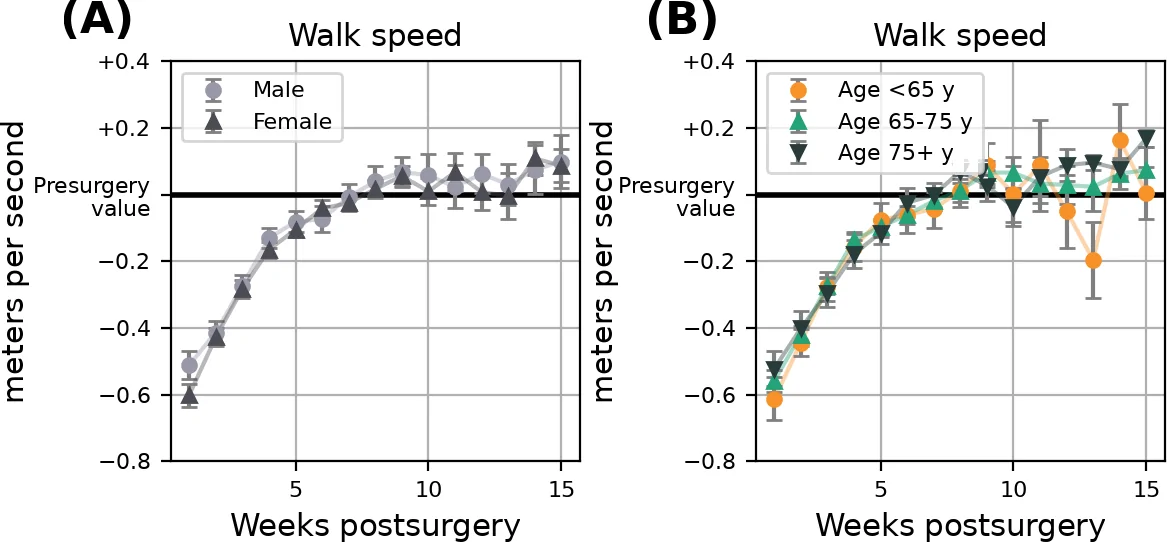
In-app recovery trajectory in walk speed after total knee arthroplasty stratified by demographic characteristics. (A) Recovery trajectories stratified by gender (male, n=158; female, n=227). (B) Recovery trajectories stratified by age (<65 y: n=76; 65‐75 y: n=200; 75+ y: n=109). The overlap between demographic subgroups indicates that patient age or gender play a limited role in the shape of the recovery trajectory at the population level (in contrast to the absolute level of gait parameters, which do differ between these subgroups). Weekly counts of patients contributing measurements in each group are given in Table S2 in Multimedia Appendix 1.

Weekly measurement counts by age and gender are shown in Table S2 in Multimedia Appendix 1.

### Recovery Trajectories by Preoperative Baseline

In addition to splitting patients by age and gender, we investigated whether preoperative baseline gait level was associated with postoperative recovery trajectories. Patients were split into 3 groups, representing the upper, middle, and lower thirds of preoperative baseline levels for each parameter. These splits were done independently for each parameter. Recovery trajectories varied greatly between the 3 groups (Figure 7 and Figure S14 in Multimedia Appendix 1). The highest-level walkers before surgery had the greatest decline in gait parameters in the week immediately following surgery and, by week 15, had reached only approximately their preoperative baseline on average. In contrast, the lowest-level walkers had a smaller decline immediately after surgery and, on average, reached well past their preoperative baseline by week 15. Kaplan-Meier and linear mixed-effects model analyses confirmed that these trajectories were significantly different for all parameters, in both THA and TKA and for in-app and background walking (Figures S15 and S16 in Multimedia Appendix 1). It should be noted that, in absolute terms, the highest-baseline group maintained better gait values at week 15 than the lowest-baseline group. However, the lowest-baseline group showed larger improvements relative to its own baseline (Figure S17 in Multimedia Appendix 1).

**Figure 7.**
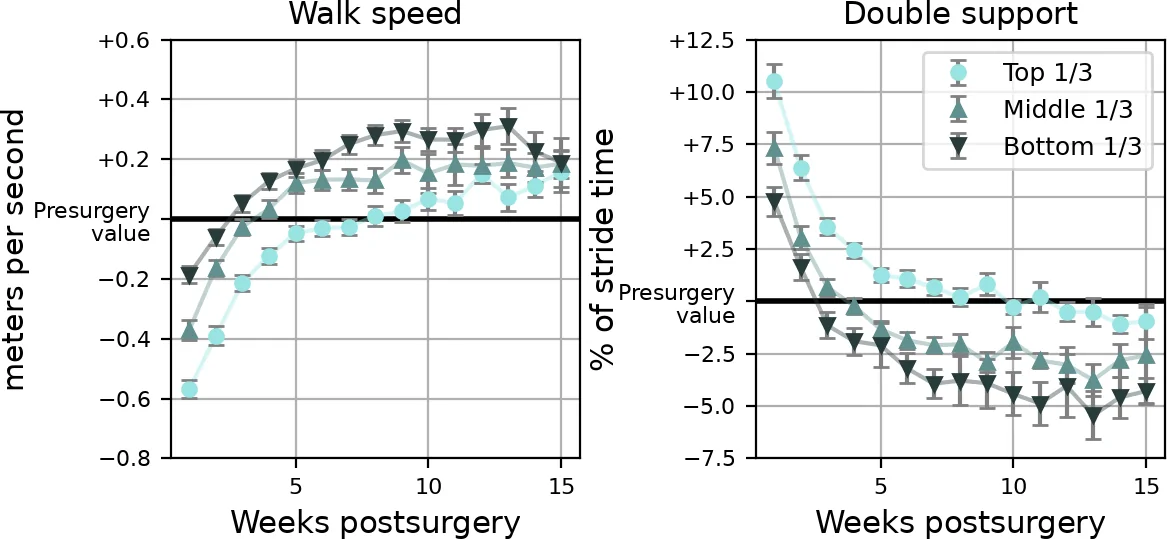
In-app recovery trajectory after total hip arthroplasty stratified by preoperative baseline level. Recovery trajectories were stratified by gait parameter values during a 14-day preoperative period; patients were split into 3 groups representing the upper, middle, and lower thirds of preoperative baseline levels for each parameter. These findings show that patients with poorer preoperative gait show larger relative gains but do not reach the absolute gait levels of higher-functioning patients, while high-functioning patients often do not return to their own baseline until later time points. Weekly counts of patients contributing measurements in each group are given in Table S3 in Multimedia Appendix 1.

## Discussion

### Principal Findings

Using longitudinal smartphone-based gait analysis, we characterized week-by-week recovery trajectories after THA and TKA across multiple spatiotemporal measures. On average, THA patients returned to their preoperative baseline by about week 4, while TKA patients did so between weeks 6 to 8, and THA patients reached a higher plateau thereafter. These differences match clinical experience and prior reports comparing postoperative mobility between procedures, which show that recovery is slower and pain persists for longer in TKA compared to THA [27,28].

A key finding is the significant difference between prompted in-app walks and passive background walking across many gait parameters. This pattern highlights the well-documented discordance between functional capacity and habitual performance [20-23], which may persist for years after surgery [29]. However, this discordance cannot be attributed solely to behavioral compensation because the 2 walking modalities are collected under fundamentally different conditions. In-app walks are performed on a flat surface under deliberate conditions, while background walks occur across heterogeneous real-world environments. Terrain type systematically influences gait parameters: energy expenditure and stride variability change substantially across surface conditions, from sidewalk to gravel to grass, and these differences are detectable by body-worn inertial sensors [30]. Walking context also matters, as indoor and outdoor walking shape distinct digital mobility signatures in ways that averaged measures can obscure [31]. Bout length is another well-established confounder of passively collected gait measures. Walking speed increases systematically with bout length, yet most real-world walking occurs in short bouts. Consequently, passive gait measures that are aggregated without accounting for bout length may not reflect gait capacity at longer sustained efforts [32,33]. This effect influences both the magnitude and reliability of extracted gait features across a range of clinical populations [33]. In the context of TKA, prearthroplasty individuals already exhibit shorter gait bouts and reduced daily walking activity compared with healthy peers [34], suggesting that passive gait measurements in this cohort are shaped not only by gait quality but also by activity patterns reflecting pain, fatigue, and behavioral avoidance.

The environmental and behavioral factors described above represent sources of structural heterogeneity in background walking that cannot be fully eliminated by algorithmic filtering. To mitigate these potential confounders, we adopted a within-person normalization approach, in which in-app and background walks were each normalized separately to their respective presurgery baselines. Any stable systematic differences between the 2 walk types arising from environmental context, walking or bout characteristics, or other persistent factors are thereby incorporated into the baseline and subtracted out. The comparison of interest is therefore not whether background and in-app parameter values differ in absolute terms: they do, and the absolute presurgery and postsurgery values for both walk types are reported here. Rather, the key comparison in this study is whether the rate and completeness of return to each condition’s own baseline differ between the in-app and background walks. Using this normalized framework, background gait consistently returns to its own presurgery level more slowly than in-app gait. We cannot fully exclude the possibility that the ecological composition of background walks shifts after surgery; for instance, early recovery may involve a greater proportion of short indoor walking bouts that differ systematically from presurgery background activity. (Because our analysis included only walking bouts lasting at least 30 s, we were unable to characterize the full distribution of walking bout lengths and contexts over the recovery period.) That being said, a shift such as this would still suggest that the patient has not returned to their regular presurgery daily walking behavior, even as their conscious gait has returned to its presurgery state. Nevertheless, the normalization approach substantially mitigates the concern that the observed divergence simply reflects a fixed structural difference between walk types, and the consistency of the pattern across the gait parameters in 2 surgical cohorts supports its robustness. Collectively, these results indicate that prompted and passively captured walks follow different recovery trajectories, and that prompted testing alone would place the point at which gait parameters stabilize earlier than the passive data indicate. What stabilization in either measure represents clinically and functionally, and which trajectory better reflects meaningful recovery, will require studies anchored to patient-reported or clinical outcomes.

Surprisingly, age- and gender-stratified trajectories appeared similar across both procedures, suggesting that the overall shape and timing of recovery, when normalized to each patient’s own baseline, are broadly robust at the population level. Although age and gender improved model fit for several parameters, the magnitude of improvement was small relative to procedure type and walk context, and neither factor was associated with differences in time to baseline in Kaplan-Meier analyses. This is at least partially at odds with prior literature, which has identified age and sex as meaningful predictors of post-TJA outcomes, though with important nuance. For TKA specifically, Pua et al [13] found older age to be among the strongest predictors of lower postoperative gait speed in a longitudinal cohort, and the large meta-analysis by Olsen et al [35], covering over 11,000 patients, identified better preoperative physical function as correlated with better 1-year physical function after TKA, though age and sex effects were inconsistent across the included studies. For THA, the predictor literature is less definitive: a systematic review by Hofstede et al [36] covering 35 studies and over 138,000 patients found that results for age, gender, and comorbidity were conflicting across studies, with preoperative function being the most consistently identified predictor. Examining both procedures jointly, Kennedy et al [37] followed 152 THA and TKA patients across the first 4 postoperative months and found that gender and baseline preoperative function both shaped early recovery trajectories, with women tending to present with greater preoperative disability and showing correspondingly different recovery curves. The apparent absence of age and gender effects in the current study should not be taken as evidence that these factors are clinically irrelevant. A key feature of our design likely attenuates them: because trajectories are normalized to each patient’s own preoperative baseline, the analysis asks whether different groups of patients recover *relative to where they started* at a different rate, not whether they reach the same absolute gait level. Since absolute gait capacity differs systematically by age and sex at baseline, normalization effectively controls for these differences, which could explain why trajectory shapes converge even when absolute gait levels do not. The finding that older patients do not take longer to return to their own preoperative baseline, while their actual gait parameter values remain lower than those of younger patients, is meaningful in its own right: it suggests that the relative recovery process is broadly consistent across demographic groups, even if absolute functional levels diverge.

In contrast, recovery trajectories differed substantially by preoperative gait level. Patients with the poorest preoperative baseline gait performance exhibited the largest improvement relative to baseline, likely reflecting greater room for improvement. In contrast, patients with higher preoperative baseline gait performance showed smaller relative gains, likely because they began closer to their functional ceiling. Nevertheless, when postoperative gait was evaluated using absolute rather than baseline-normalized values, these higher-functioning patients still achieved higher absolute postoperative gait levels. Thus, relative improvement and absolute gait performance capture distinct aspects of recovery and should be interpreted together. These phenomena are well documented in the TJA literature. Across TKA studies, preoperative gait function, quadriceps strength, and overall physical function have consistently predicted postoperative performance-based outcomes, gait-speed recovery, and 1-year physical function, while lower baseline function and higher BMI have been associated with poorer absolute outcomes [13,35,38,39]. At the same time, patients with higher preoperative gait function may show smaller relative gains or even early functional decline, consistent with our finding that the highest-baseline group returned only approximately to its own preoperative level by week 15 [38]. Similar patterns have been reported after THA: patients with worse preoperative function often improve more, but do not reach the absolute postoperative function achieved by those who started at a higher level [36,37].

Taken together, the results of this study support recovery targets that are individualized to each patient’s baseline function rather than defined by a single absolute threshold. Whether the difference in baseline is due to demographic considerations or individual variation, valuable clinical information is lost when recovery is evaluated solely against population-based gait parameter values. A patient who walks slowly before surgery and returns to that level has a different clinical interpretation than a higher-functioning patient who fails to recover to their own preoperative level. Thus, both the absolute level of gait performance and the trajectory of recovery relative to baseline provide distinct and complementary clinical information.

### Limitations

Within this observational study, we did not have access to and therefore did not control for all factors known to affect recovery, such as surgical approach, implant type, perioperative protocols, rehabilitation protocols, pain level, or comorbidities [40]. Further, no anthropometric normalization was performed (eg, normalizing stride length by patient height) because such data were also unavailable. Age was available for patients, and we grouped patients using clinically meaningful age ranges rather than equal-sized groups. This allowed us to compare age categories that are more interpretable clinically, but the resulting uneven group sizes should be considered when interpreting the age-stratified analyses.

We chose to use the return to preoperative baseline as the primary metric in this study, for the reasons described above. However, because most patients expect to achieve better function and/or less pain than they experienced in the weeks before surgery, a return to baseline should not be conflated with full recovery. In this study, the data did not allow for an analysis focused on, for example, reaching a clinically meaningful level above the presurgery baseline, since many patients had not exceeded their baseline by a noticeable margin by week 15. Future studies in this vein should aim to extend the timeline of data collection so as to be able to directly assess recovery beyond baseline.

Time to return to baseline, as calculated with Kaplan-Meier analysis, used the first week in which the patient reached their presurgery level. This definition may overestimate the speed of recovery in patients whose first crossing of baseline was transient. Functional recovery after TJA can fluctuate because of pain, fatigue, activity level, or other short-term factors, and a patient who reaches baseline in 1 week may fall below baseline in a subsequent week. The Kaplan-Meier results should therefore be interpreted as time to first observed return to baseline, rather than time to sustained recovery beyond baseline.

While smartphone-based data collection vastly increases the number of subjects it is possible to include in these types of studies, one downside compared with using dedicated hardware is that there is much more missing data within a subject’s dataset. Adherence and phone-carriage behaviors (whether people carry their phones in their pants pockets while walking) influence who contributes data over time, and environmental variability can affect background measurements as well. For this reason, we do not see multiple passive walking bouts per day in many subjects, whereas previous studies using more dedicated measurement systems do find walking bouts at that frequency, although in cohort sizes more than an order of magnitude smaller than our study [31,41].

We summarized cohorts without imputing missing weeks, so missingness could relate to recovery status. Further, diminishing sample sizes in later weeks (particularly for in-app walks) reduce statistical power and make late-week estimates highly sensitive to outliers. This analysis may also be subject to survivorship bias because patients who continued contributing data at later weeks may not be representative of the original cohort. In particular, if continued participation is related to recovery status, the observed results may reflect both true recovery and the changing composition of the patients who remained under observation. Therefore, the values at later weeks should be interpreted with caution.

Passive data collection depends on the patient carrying their smartphone in a pants pocket, which may introduce selection bias. Our filtering methods for removing walks in which the smartphone was outside the pants pocket could also have failed to filter some such walks, introducing noisy and unreliable data into the dataset. In addition, as discussed above, passively collected data reflect walking undertaken in a variety of conditions, which makes such data inherently different from prompted data collection. This fact is mitigated by our normalization of the in-app and background data separately to their respective presurgery levels, but there could still be an effect; for example, if the types of passive walks undertaken after surgery differ systematically from those taken before surgery. Finally, our analysis ended at 15 weeks after surgery, so later improvements or setbacks were not captured.

### Conclusions and Implications for Future Research

Despite these limitations, this study provides a robust quantitative characterization of gait-parameter recovery trajectories in the first 15 weeks after TJA at weekly resolution and demonstrates the power of smartphone-based gait analysis to generate longitudinal datasets at scale. The differences in in-app and background walking that we observed suggest that clinical decisions, such as when to taper walking aids or advance activity, could benefit from incorporating passive background metrics rather than relying solely on prompted tests. However, studies that directly assess these questions are needed before any concrete clinical recommendations can be made. Further analyses of this dataset could quantify within-week heterogeneity, identify early signals associated with delayed recovery, and cluster longitudinal trajectories to define distinct recovery trajectory profiles. Defining and validating these profiles and linking them to clinical correlates could support clearer counseling and targeted interventions. Beyond this dataset, future work should extend follow-up, link trajectories to complications and PROMs, and incorporate factors such as BMI, height, and rehabilitation exposure.

## Supplementary material

10.2196/97657 Multimedia Appendix 1 Supplementary figures and tables supporting the analysis of postoperative gait recovery after total hip and total knee arthroplasty.

10.2196/97657 Multimedia Appendix 2 Aggregate (group-level) mean values of the 9 gait parameters analyzed in this study, provided for each combination of procedure (total hip arthroplasty, total knee arthroplasty), walk type (in-app, background), and week (preoperative baseline through week 15 postsurgery).

## Abbreviations

EMM: estimated marginal mean
IMU: inertial measurement unit
PROM: patient-reported outcome measure
THA: total hip arthroplasty
TJA: total joint arthroplasty
TKA: total knee arthroplasty
