# Supplementary material for: Real-World Functional Gait Recovery Trajectories After Total Hip and Total Knee Arthroplasty via Smartphone Sensors: Observational Longitudinal Study
Source: JMIR Mhealth Uhealth. 2026 Oct 30;14:e97657. doi: 10.2196/97657 (PMC13721847; doi:10.2196/97657)
Supplement: Supplementary file 1 [file mhealth-v14-e97657-s001.pdf]

Supplemental Figures & Tables

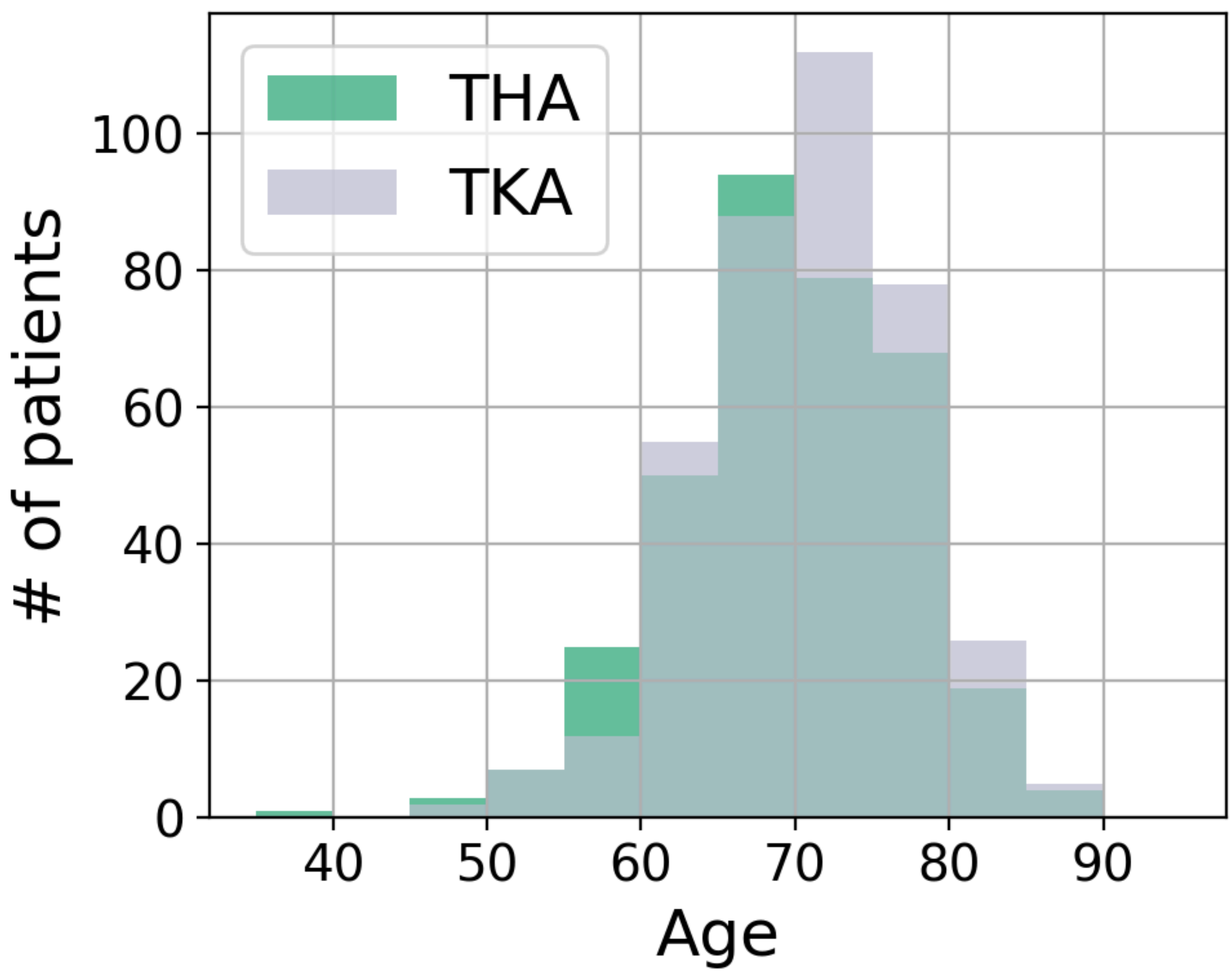

Figure S1. Age distributions of THA and TKA cohorts.

a

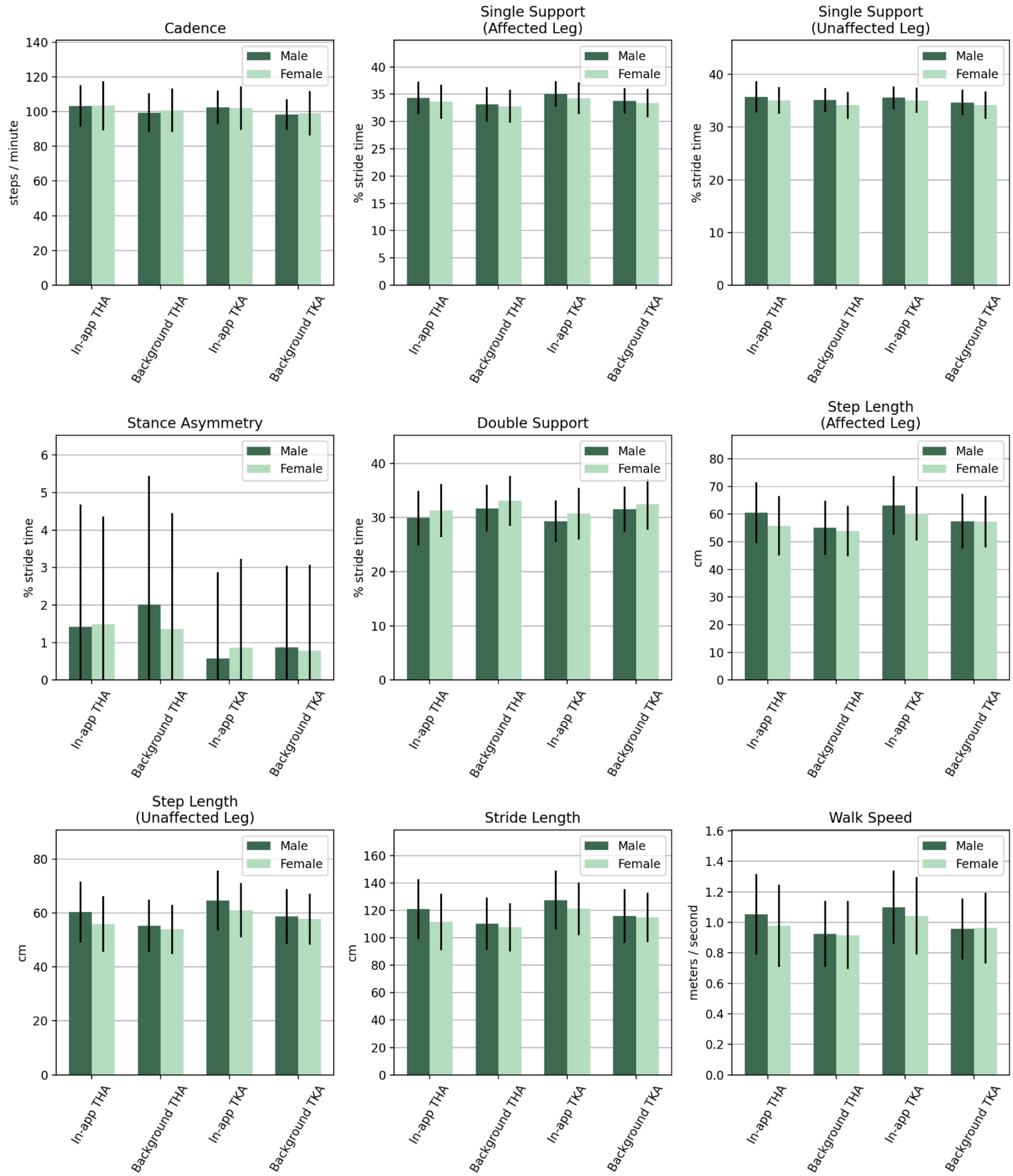

Figure S2. Preoperative baseline values of gait stratified by a. gender and b. age. Error bars represent standard deviation.

b

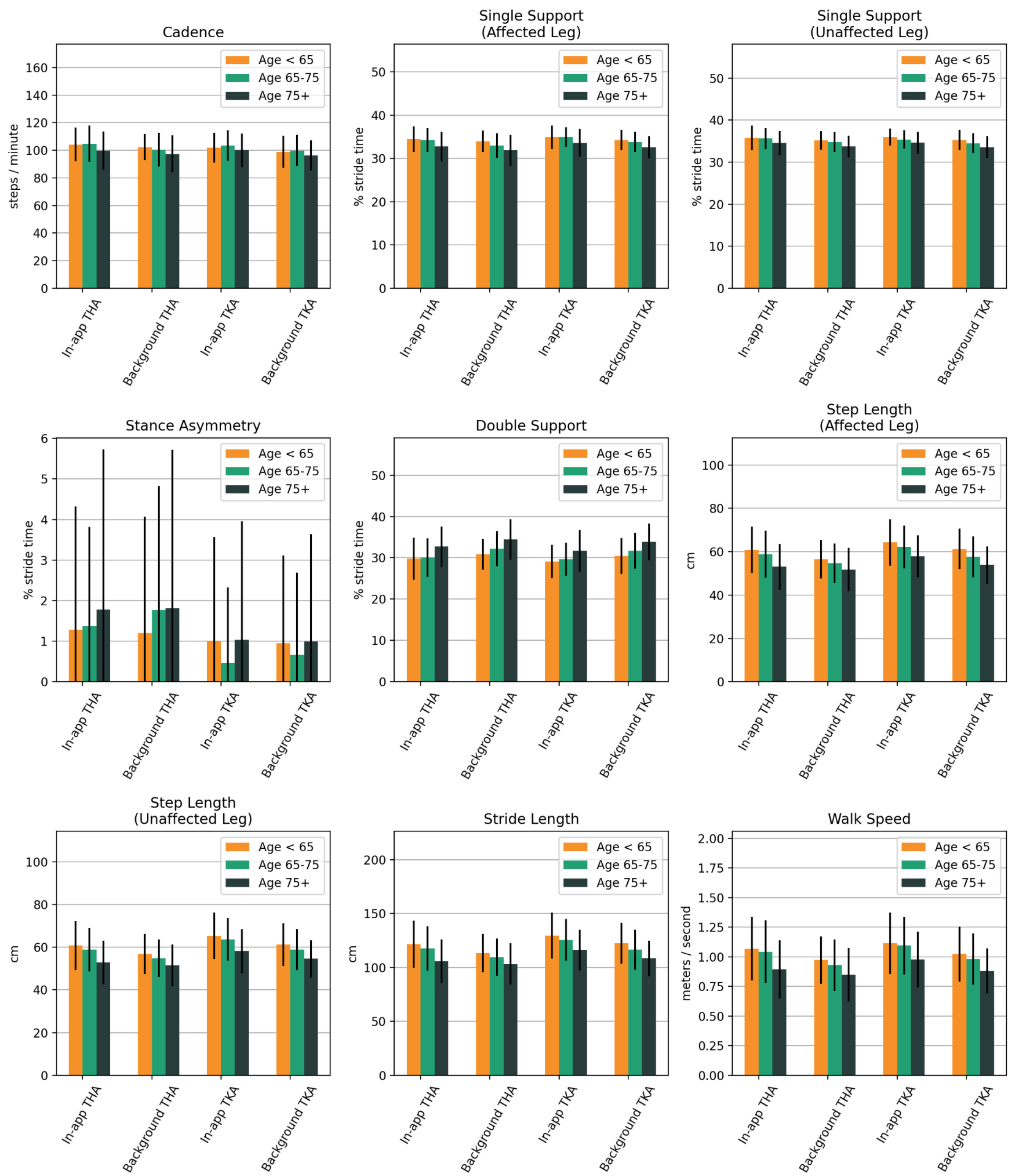

Figure S2 (continued).

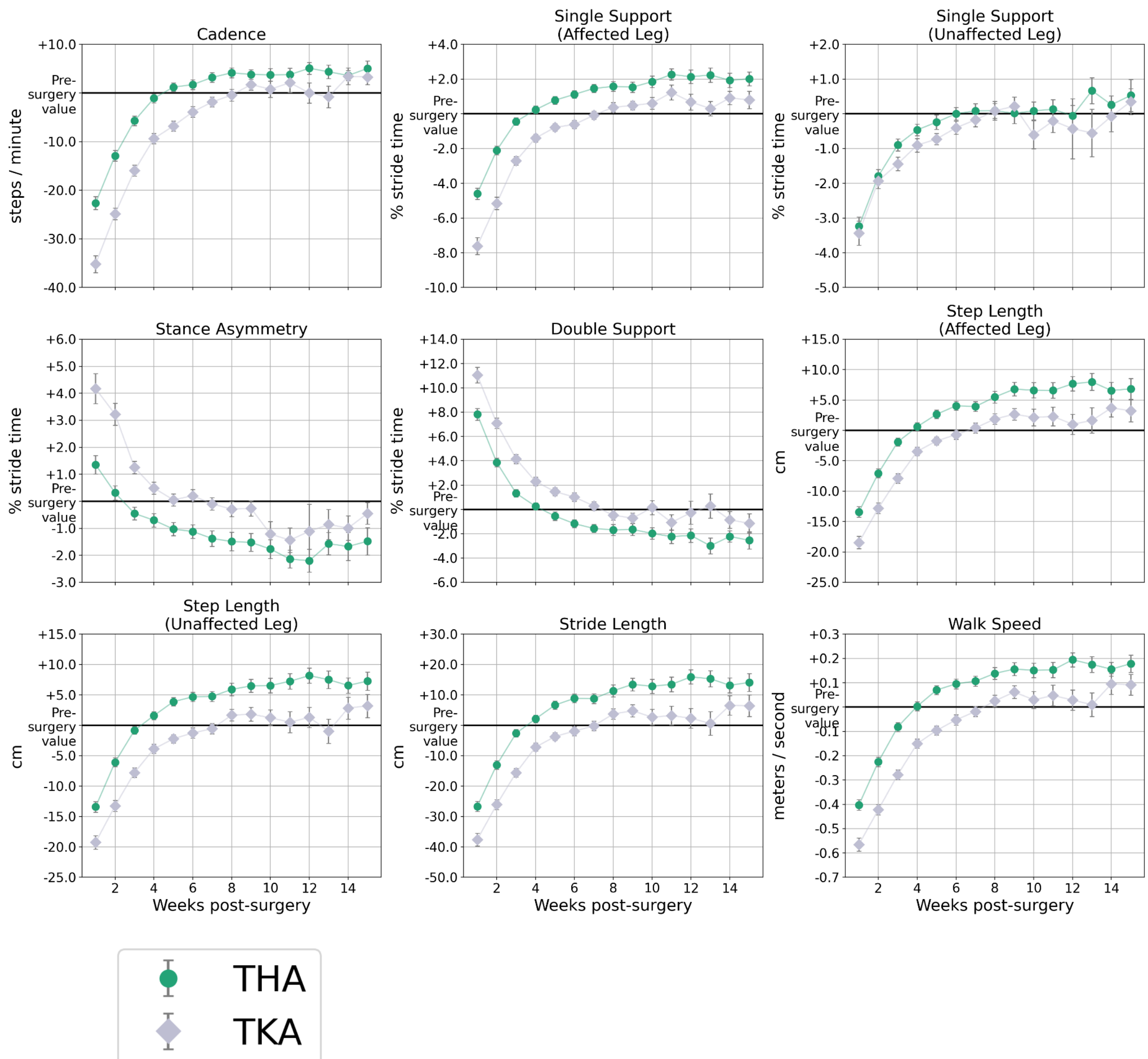

Figure S3. Recovery of in-app gait parameters after surgery in THA vs. TKA. Each data point represents the mean change from all patients' preoperative baselines for that postoperative week; error bars show standard error. Positive values indicate increases relative to baseline. Improvement corresponds to increases in cadence, step length, stride length, and walk speed, and to decreases in double support time and stance asymmetry. “Affected” refers to the operated limb; “unaffected” refers to the contralateral limb.

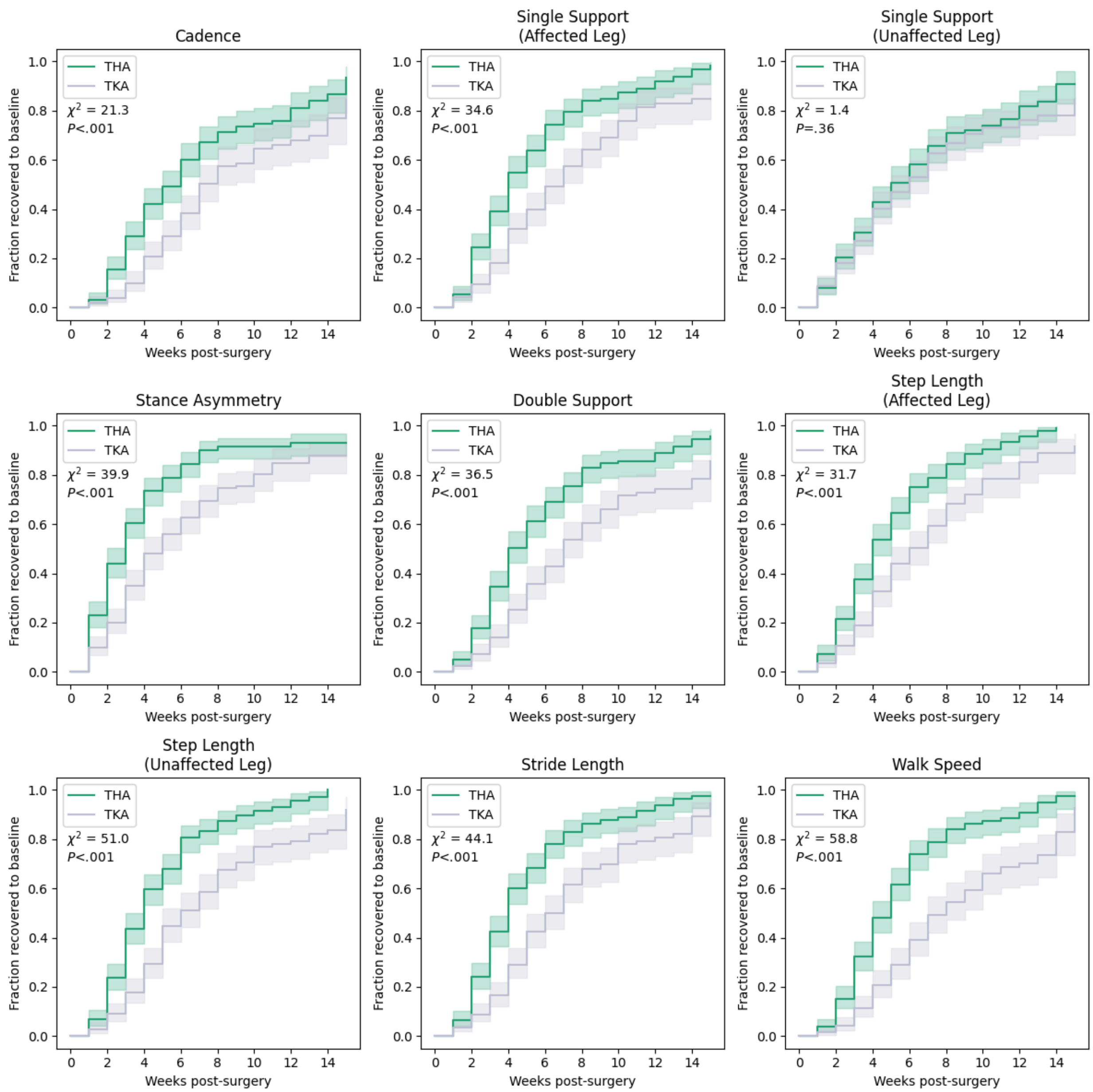

Figure S4. Kaplan-Meier analysis of THA vs. TKA. The curves show the fraction of patients whose average parameter value crossed their own pre-surgery gait level by each postoperative week. Shaded bands indicate 95% CIs. Each plot shows the χ^2 test statistic (df=1) and FDR-corrected P value of a log-rank test to determine if the curves differ significantly.

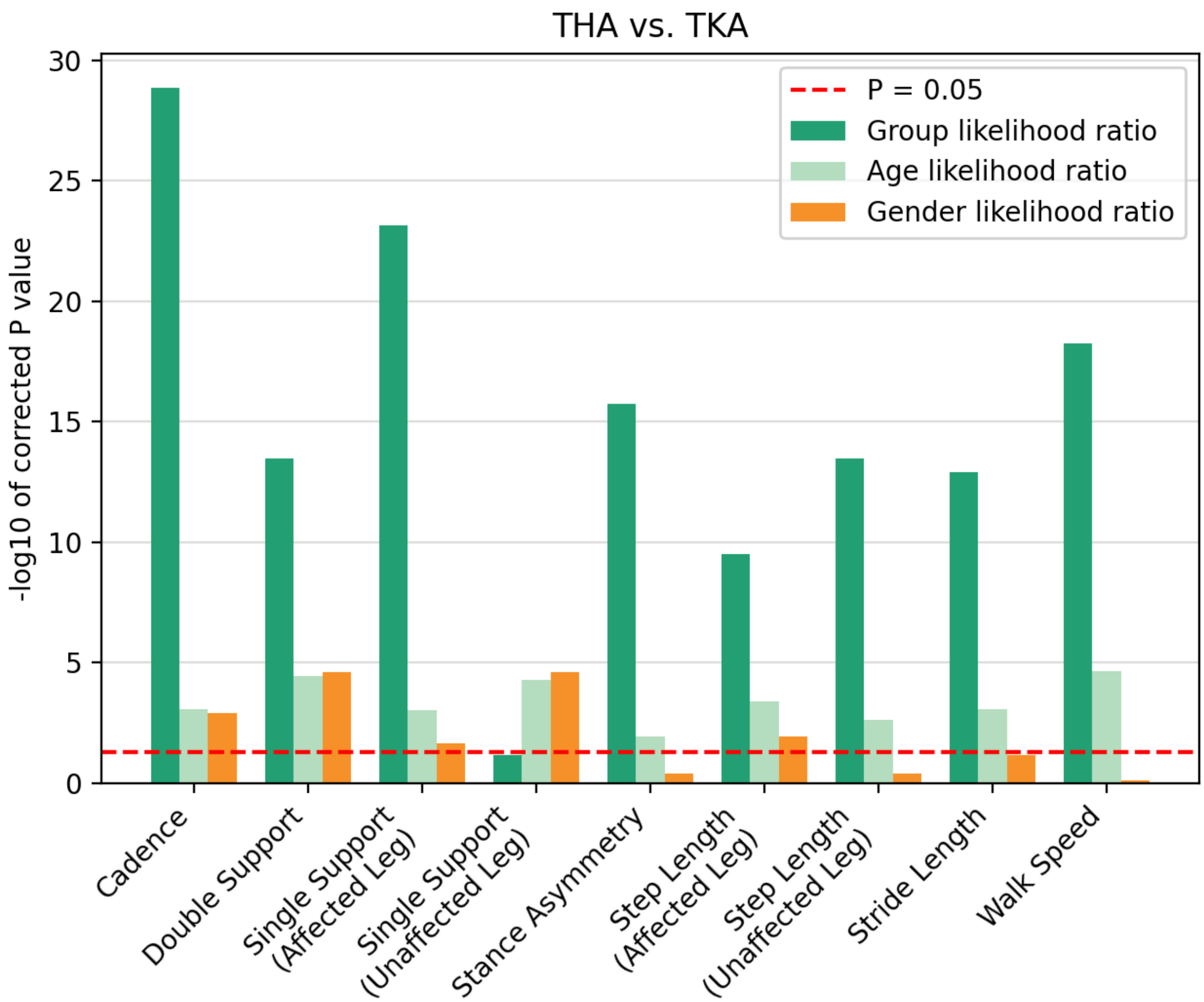

Figure S5. Likelihood ratio test (LRT) P values from nested linear mixed-effects models of THA vs. TKA. Models evaluated the contribution of key fixed effects across nine gait parameters. For each gait parameter, a full model including group (THA vs. TKA), age, and gender (and the shared time/recovery-curve specification and random-effects structure) was fit by maximum likelihood. Three reduced models were then fit, each omitting one term (condition, age, or gender) while keeping all other terms and the random-effects structure identical. The plotted P values correspond to the LRT comparing each reduced model to its matched full model, quantifying the evidence that the removed term improves model fit for each parameter. All P values were adjusted for multiple testing using the Benjamini-Hochberg FDR method.

a

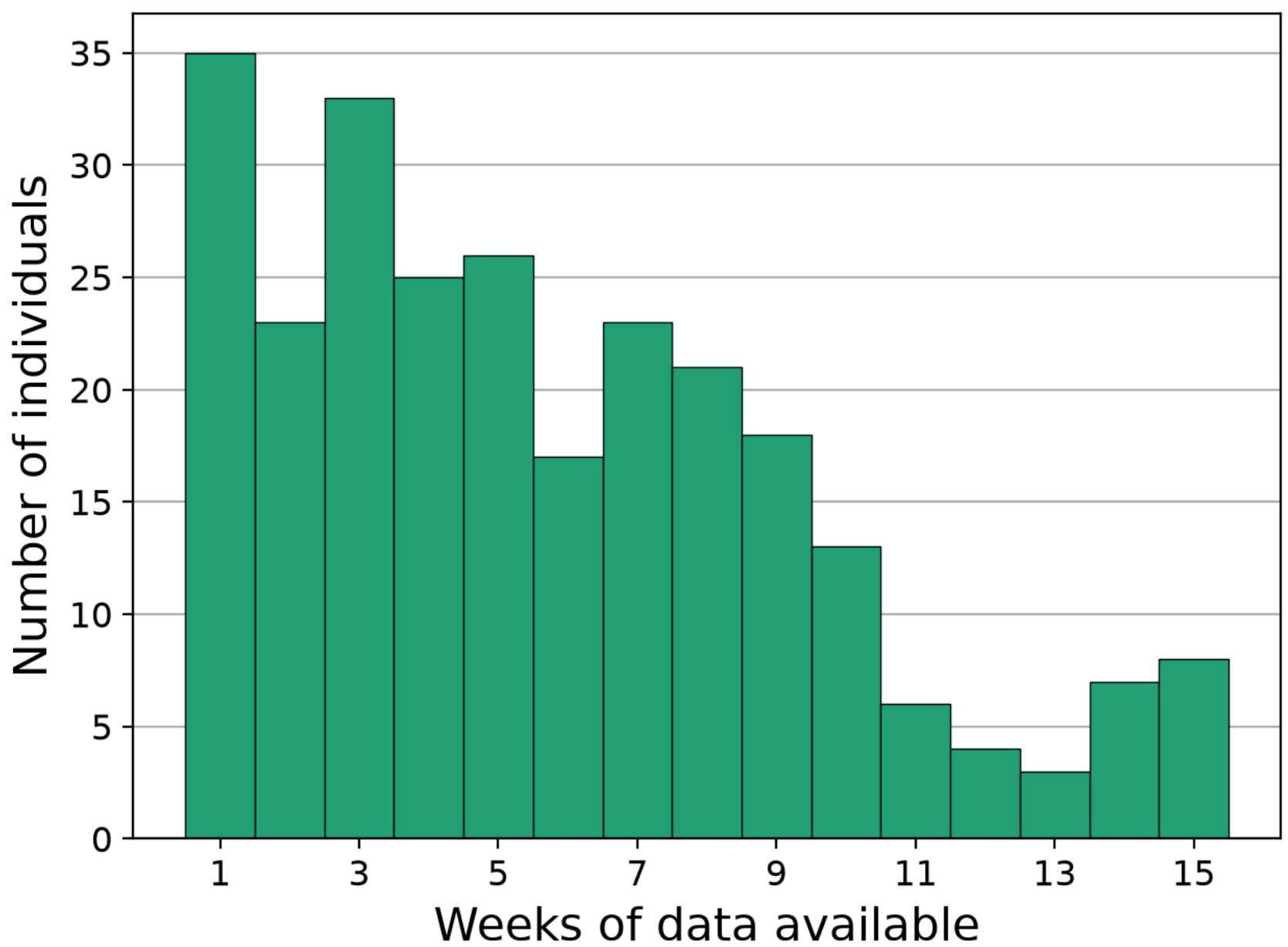

b

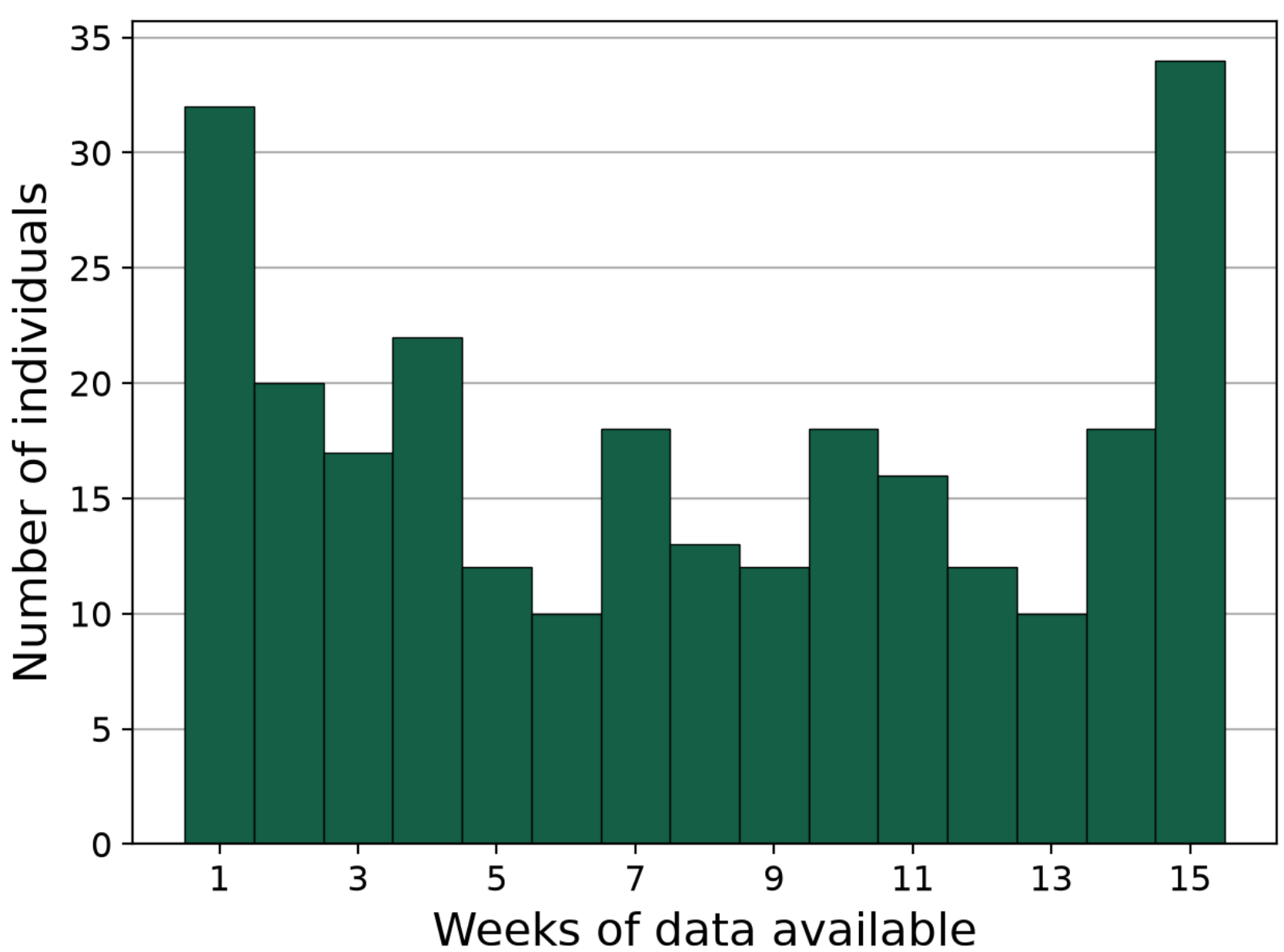

Figure S6. Distribution of the number of postoperative weeks with available data per patient. Each histogram shows how many patients (y-axis) contributed gait data in a given number of distinct postoperative weeks (x-axis) in the 15 weeks after surgery. **a**. In-app, THA. **b**. Background, THA. **c**. In-app, TKA. **d**. Background, TKA. Most patients contributed data in only a subset of postoperative weeks. Patients who recorded no post-surgery walks of the relevant type are not shown.

c

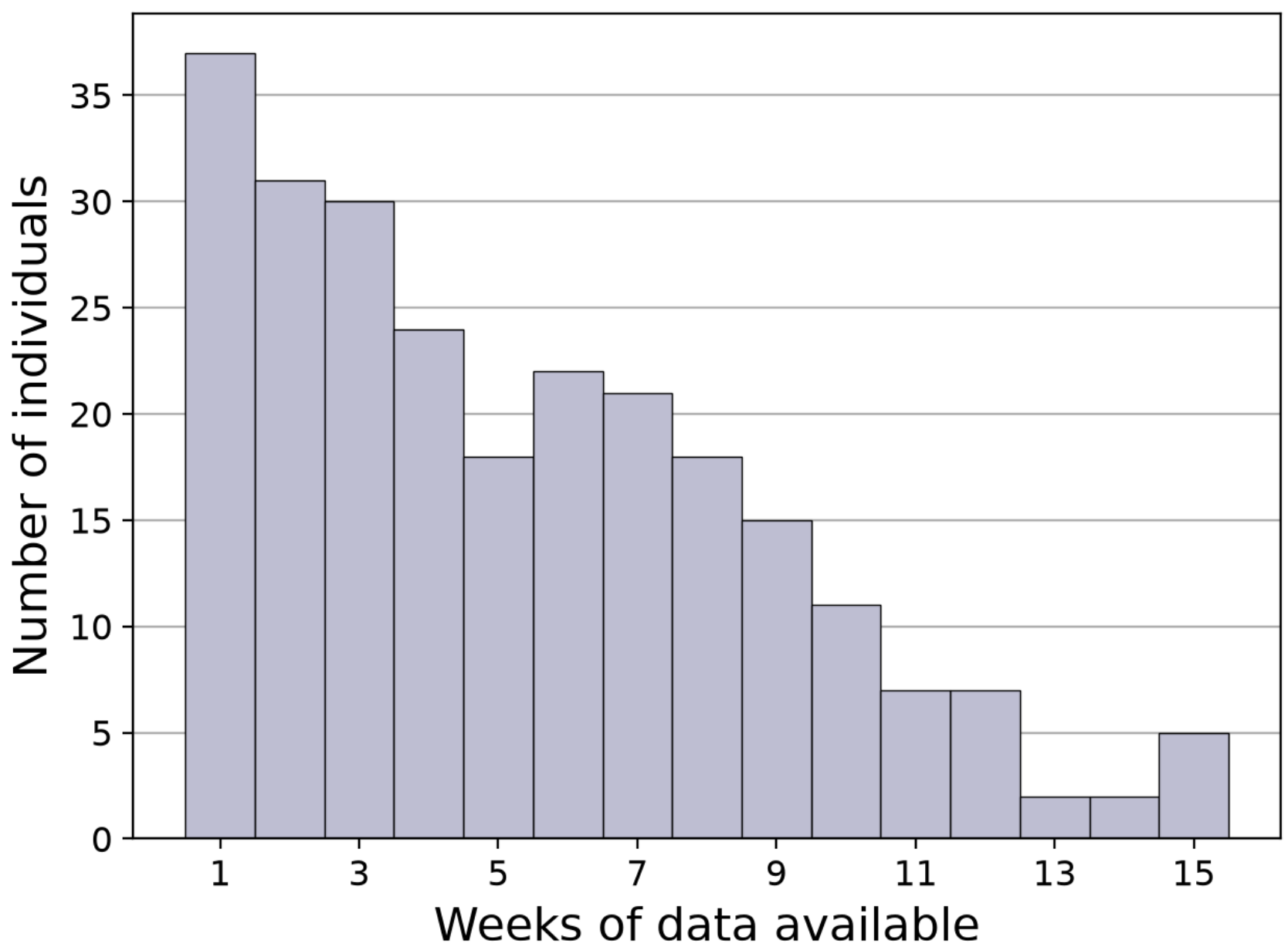

d

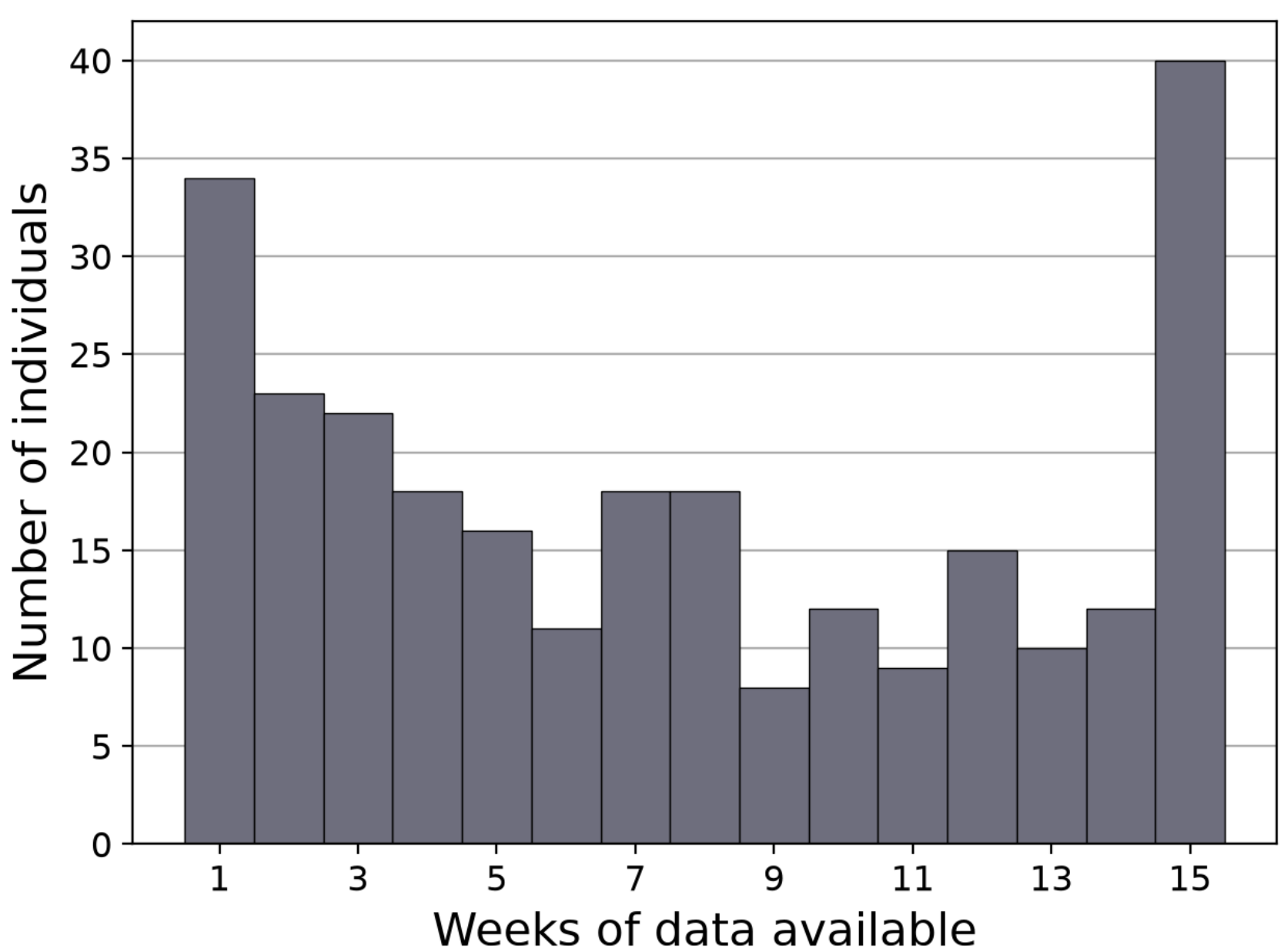

Figure S6. Distribution of the number of postoperative weeks with available data per patient (continued).

a

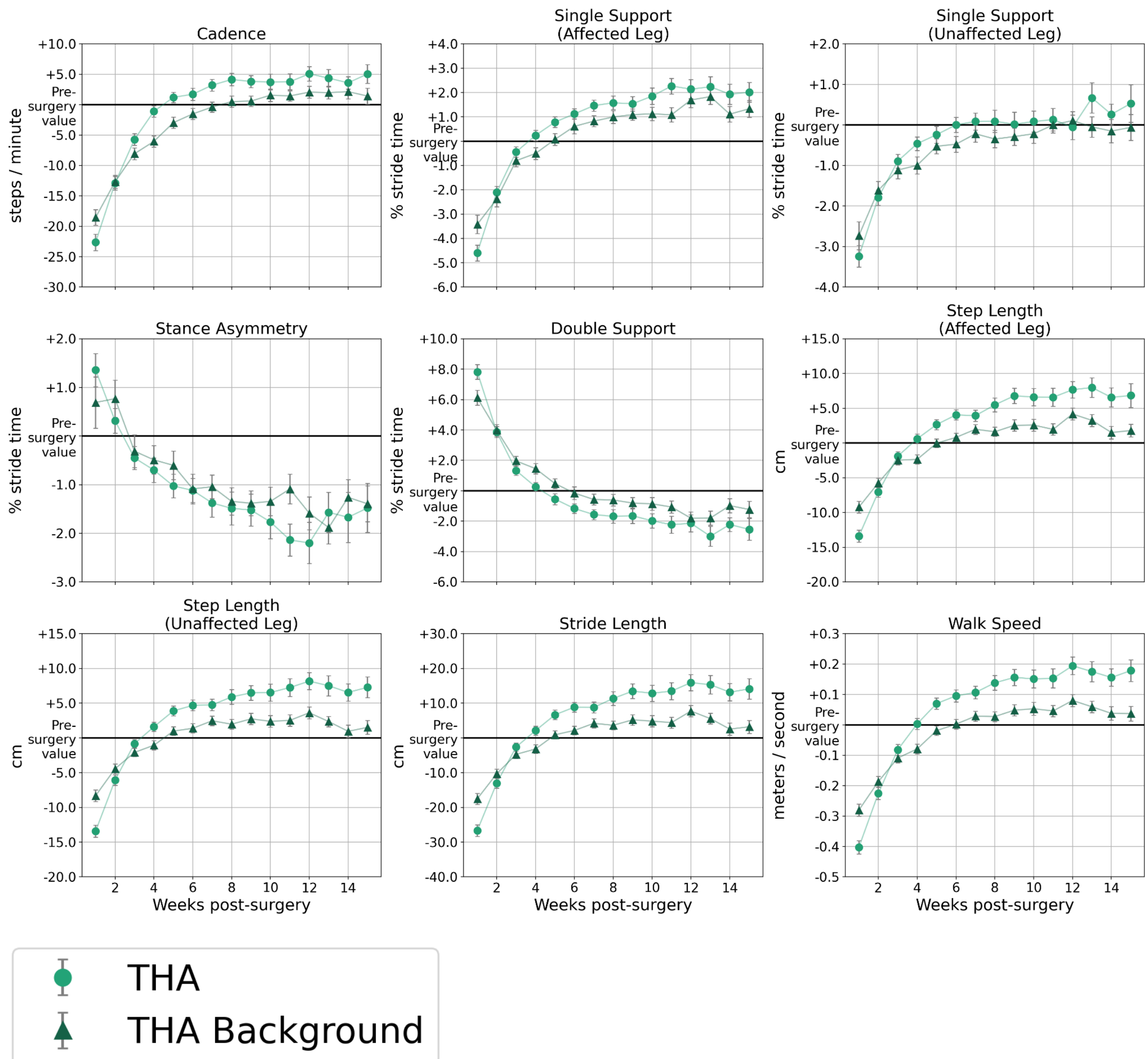

Figure S7. Recovery of in-app vs. background gait parameters after surgery. a. Recovery after THA. **b.** Recovery after TKA. Each data point represents the mean change from all patients' preoperative baselines for that postoperative week; error bars show standard error. Positive values indicate increases relative to baseline. Improvement corresponds to increases in cadence, step length, stride length, and walk speed, and to decreases in double support time and stance asymmetry. "Affected" refers to the operated limb; "unaffected" refers to the contralateral limb.

b

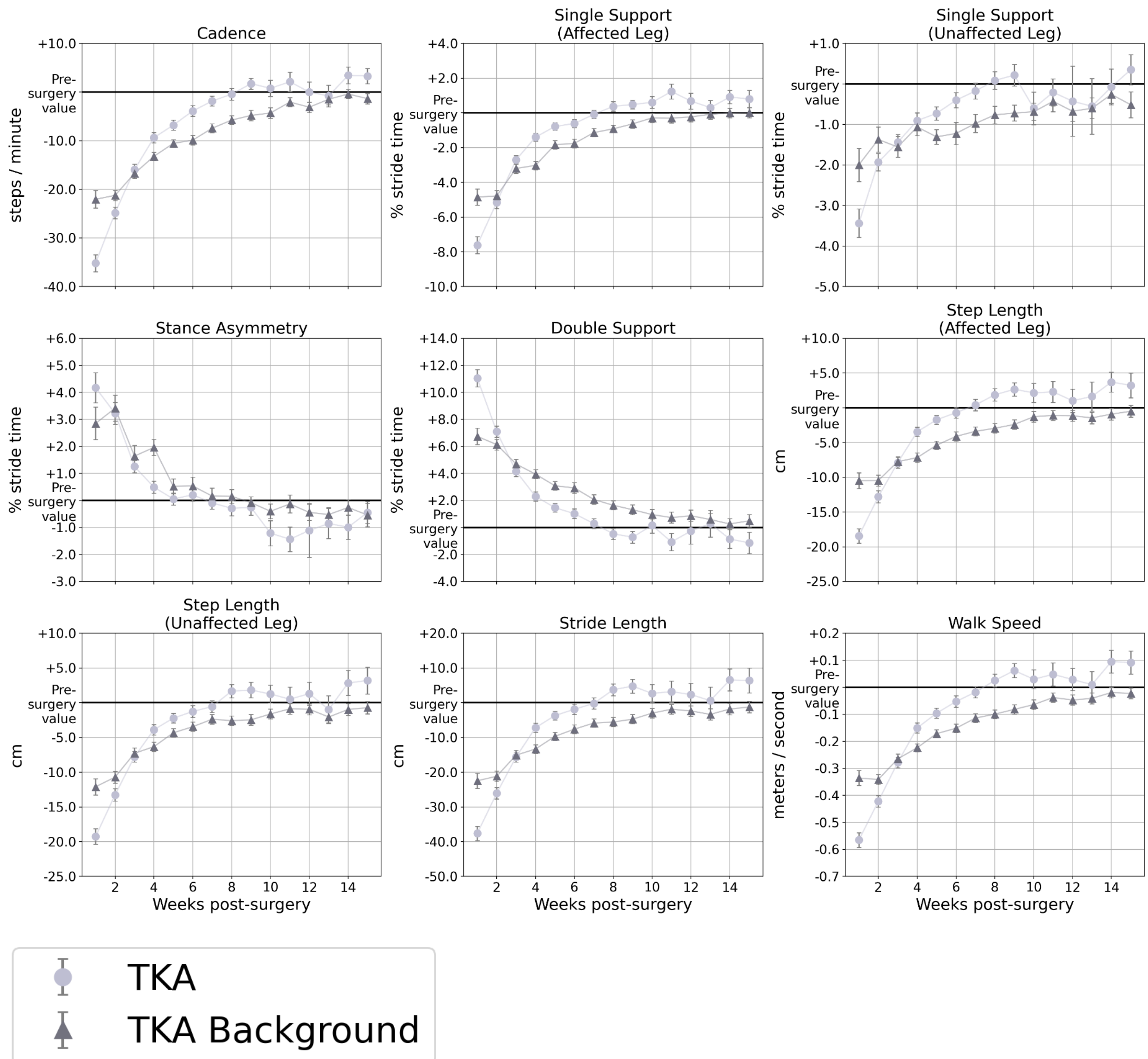

Figure S7 (continued).

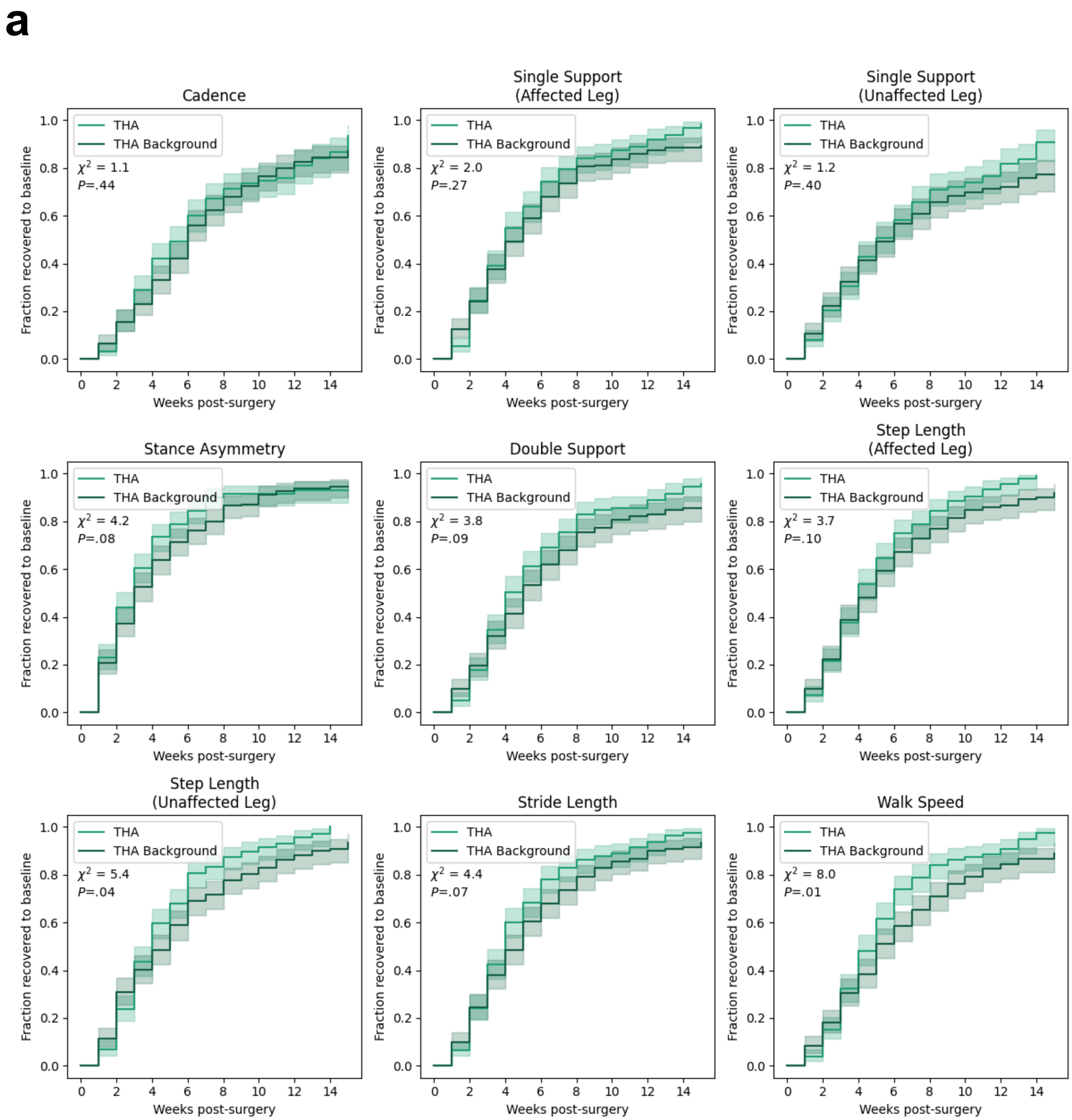

Figure S8. Kaplan-Meier analysis of in-app vs. background recovery. The curves show the fraction of patients whose average parameter value crossed their own pre-surgery gait level by each postoperative week. Shaded bands indicate 95% CIs. Each plot shows the χ^2 test statistic (df=1) and FDR-corrected P value of a log-rank test to determine if the curves differ significantly. **a.** In-app vs. background after THA. **b.** In-app vs. background after TKA.

b

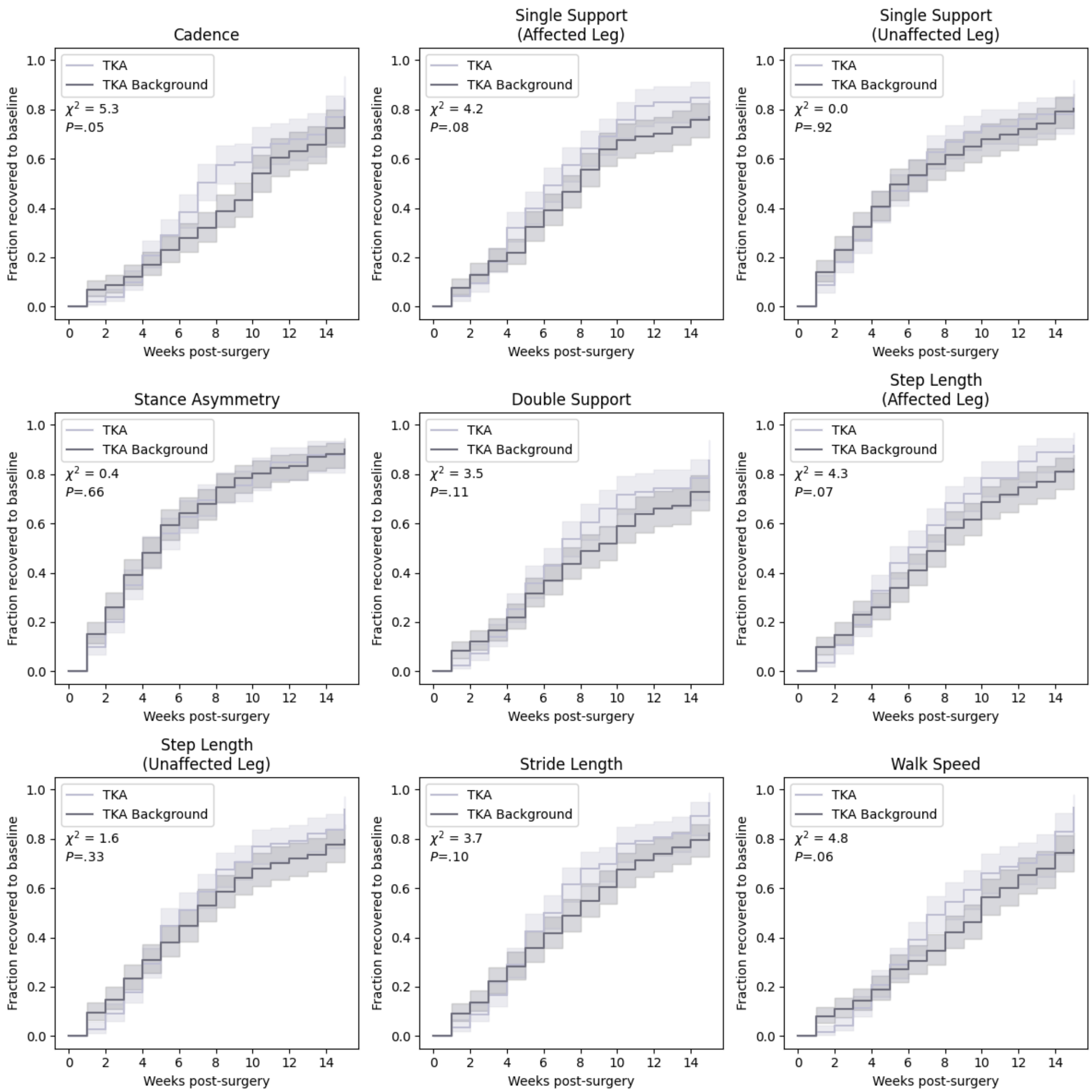

Figure S8 (continued).

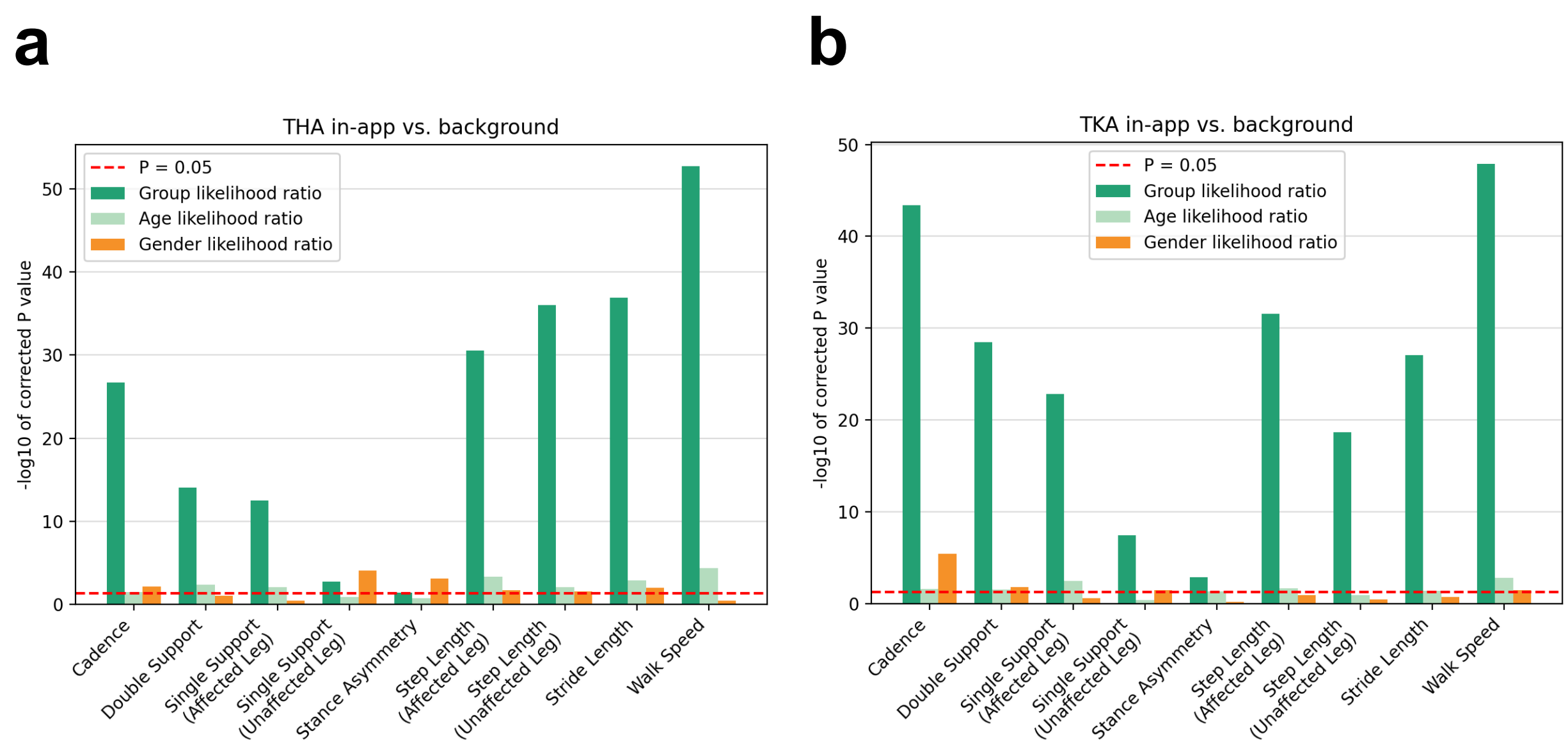

Figure S9. Likelihood ratio test (LRT) P values from nested linear mixed-effects models of in-app vs. background. Models evaluated the contribution of key fixed effects across nine gait parameters. For each gait parameter, a full model including group (in-app vs. background), age, and gender (and the shared time/recovery-curve specification and random-effects structure) was fit by maximum likelihood. Three reduced models were then fit, each omitting one term (condition, age, or gender) while keeping all other terms and the random-effects structure identical. The plotted P values correspond to the LRT comparing each reduced model to its matched full model, quantifying the evidence that the removed term improves model fit for each parameter. All P values were adjusted for multiple testing using the Benjamini-Hochberg FDR method. **a.** In-app vs. background after THA. **b.** In-app vs. background after TKA.

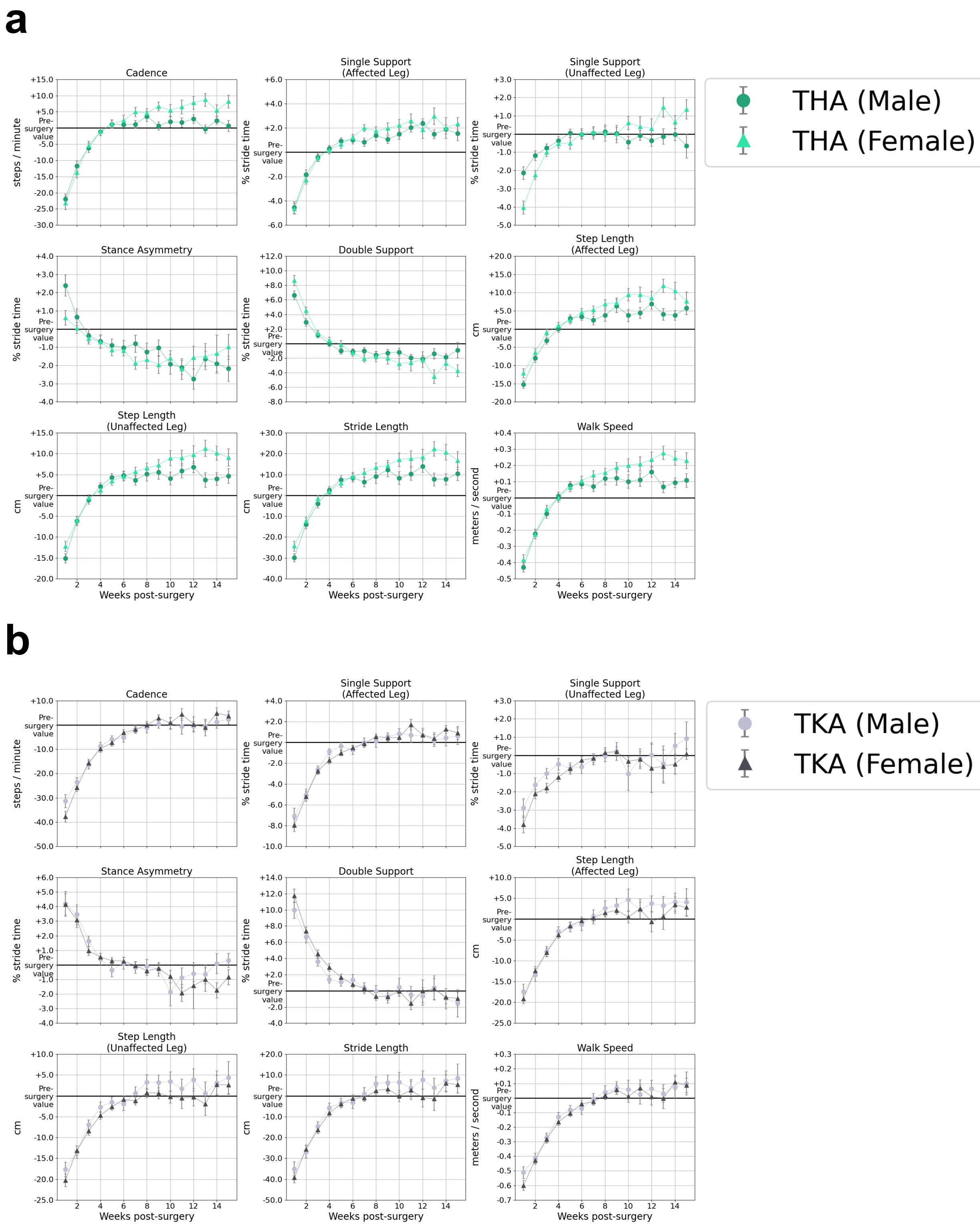

Figure S10. In-app recovery stratified by gender. a. Recovery after THA (male: *n*=153; female: *n*=197). **b.** Recovery after TKA (male: *n*=158; female: *n*=227).

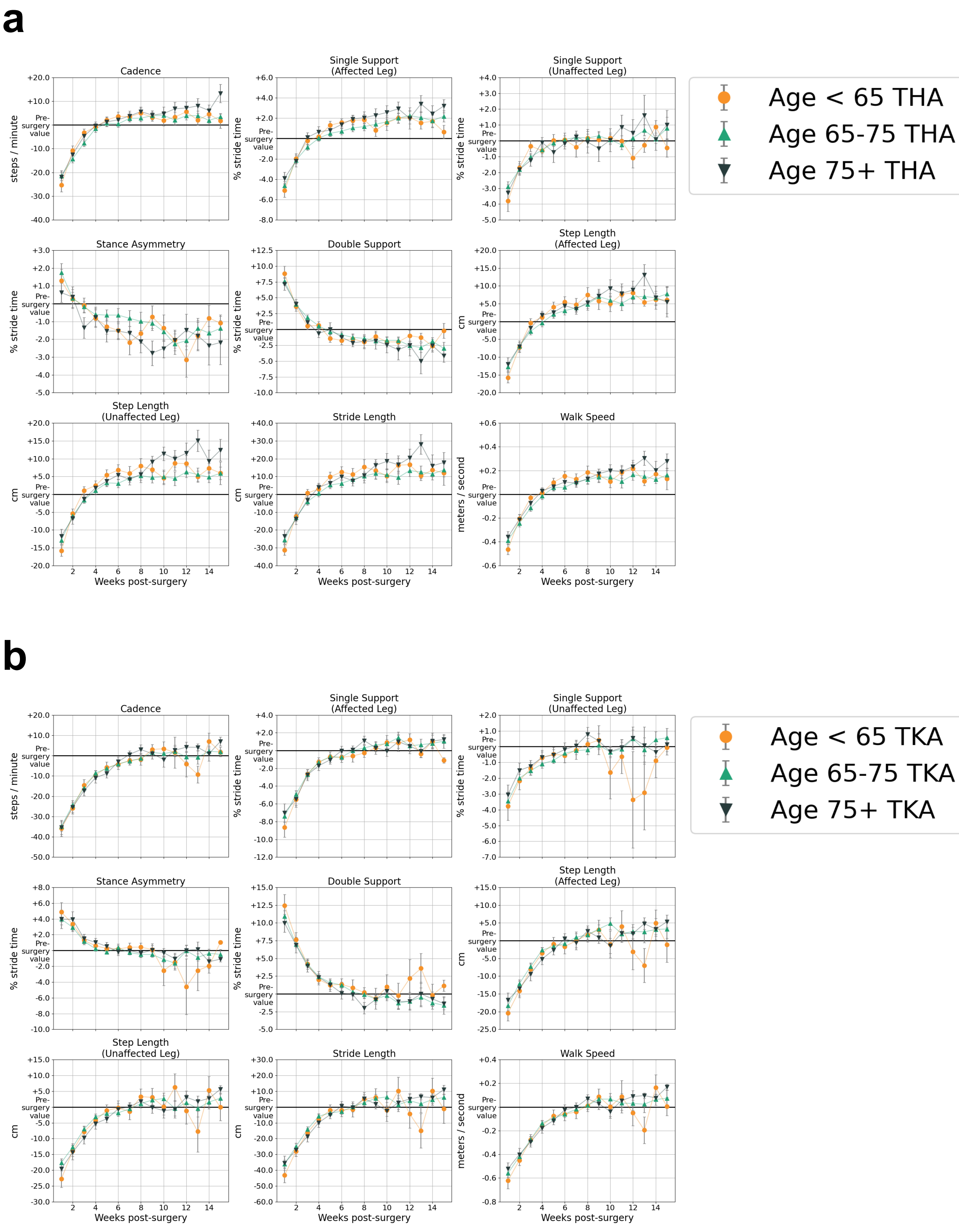

Figure S11. In-app recovery stratified by age. a. Recovery after THA (<65: *n*=86; 65–75: *n*=173; 75+: *n*=91). **b.** Recovery after TKA (<65: *n*=76; 65–75: *n*=200; 75+: *n*=109).

a

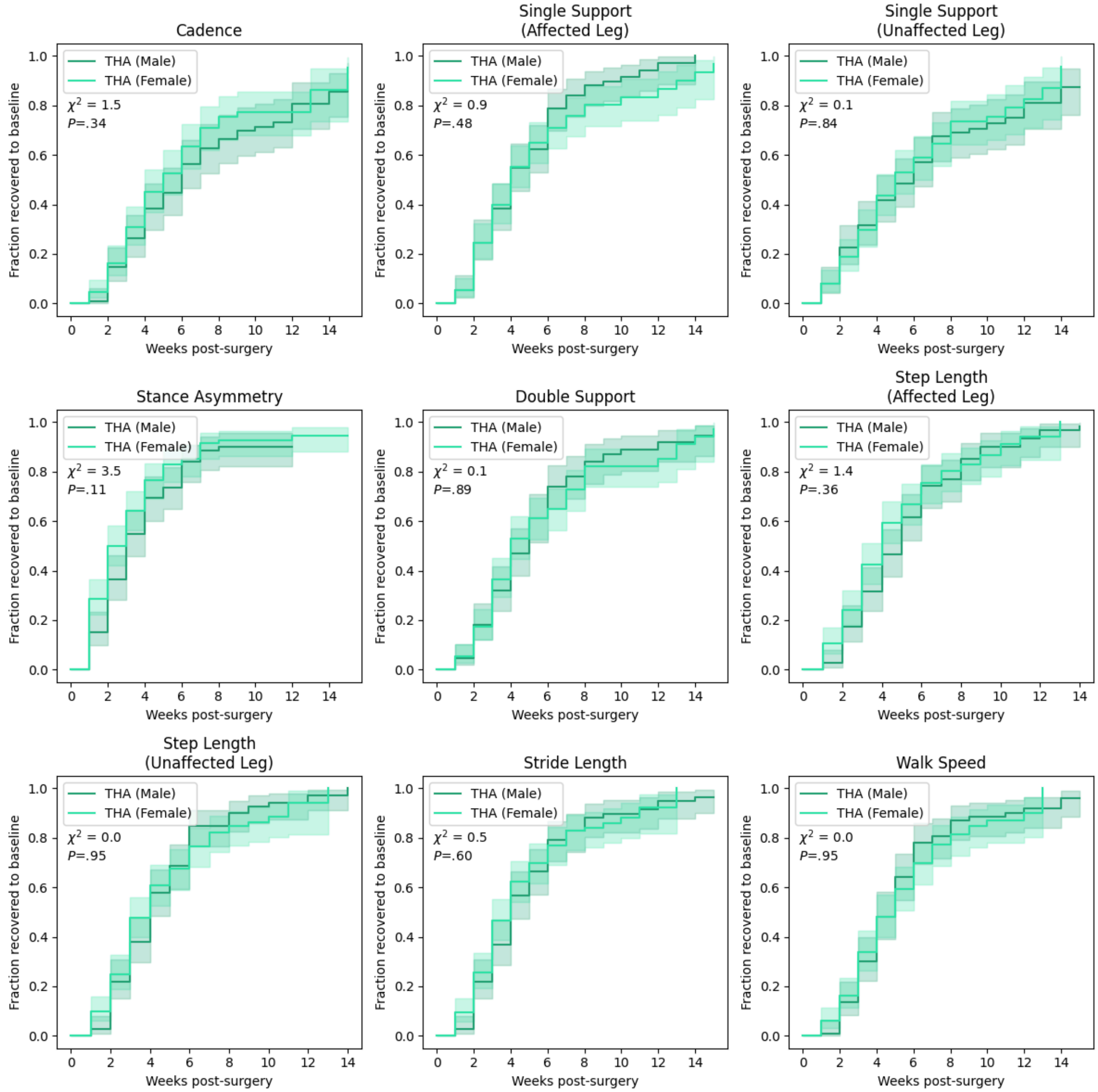

Figure S12. Kaplan-Meier analysis stratified by gender. The curves show the fraction of patients whose average parameter value crossed their own pre-surgery gait level by each postoperative week. Shaded bands indicate 95% CIs. Each plot shows the χ^2 test statistic (df=1) and FDR-corrected P value of a log-rank test to determine if the curves differ significantly. **a.** THA in-app. **b.** TKA in-app.

b

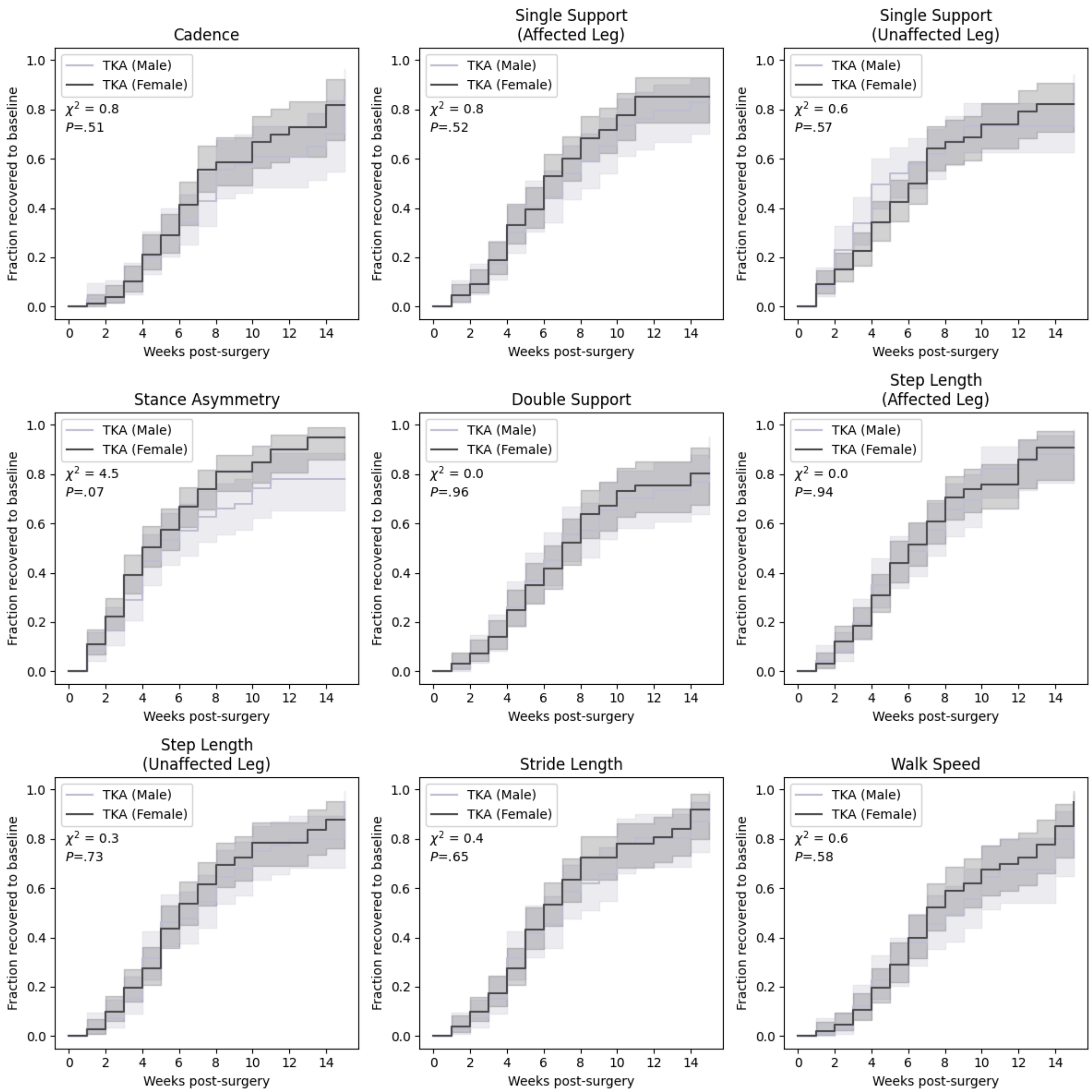

Figure S12 (continued).

a

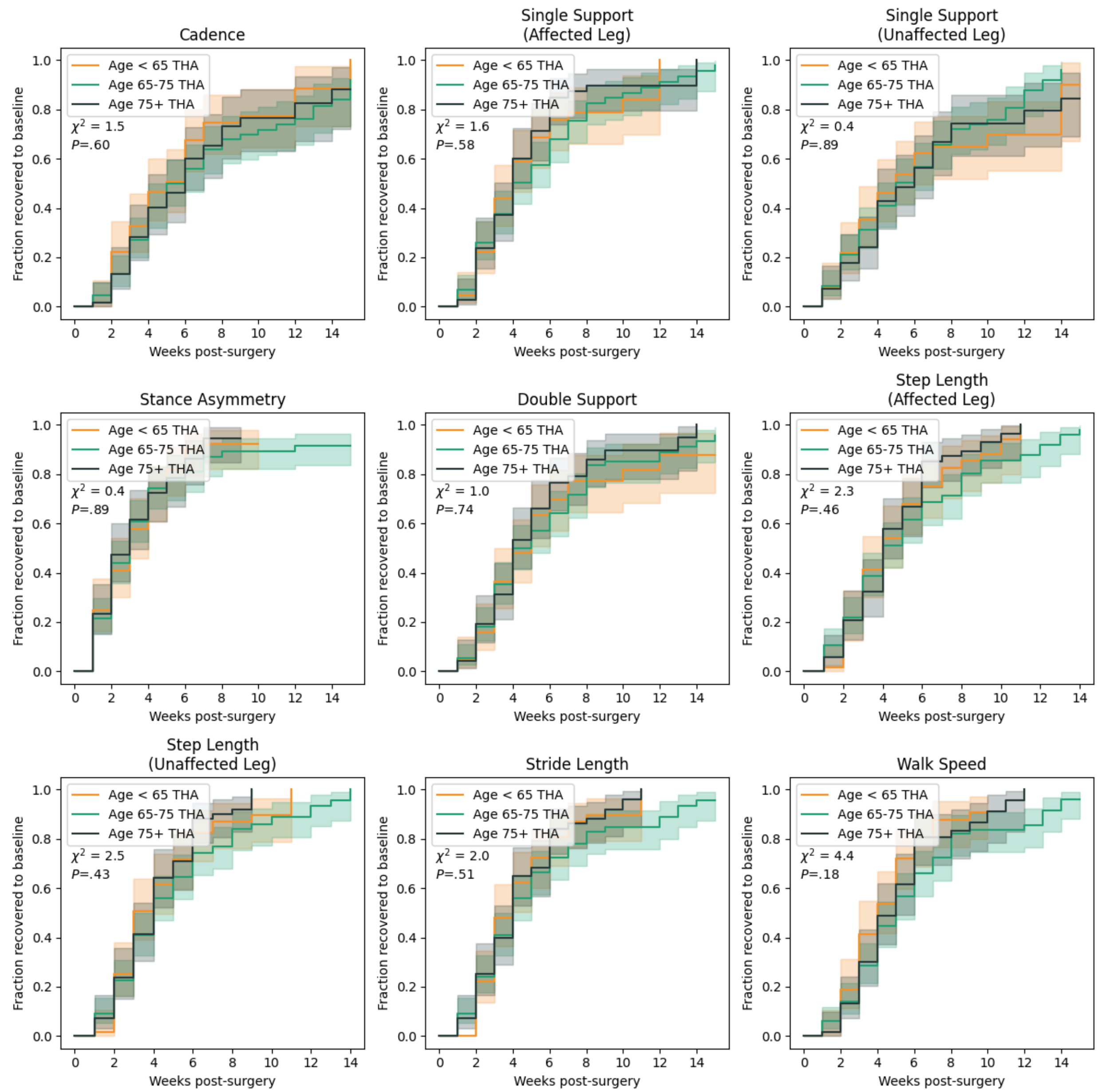

Figure S13. Kaplan-Meier analysis stratified by age. The curves show the fraction of patients whose average parameter value crossed their own pre-surgery gait level by each postoperative week. Shaded bands indicate 95% CIs. Each plot shows the **χ²** test statistic (df=2) and FDR-corrected P value of a multivariate log-rank test to determine if the curves differ significantly. **a.** THA in-app. **b.** TKA in-app.

b

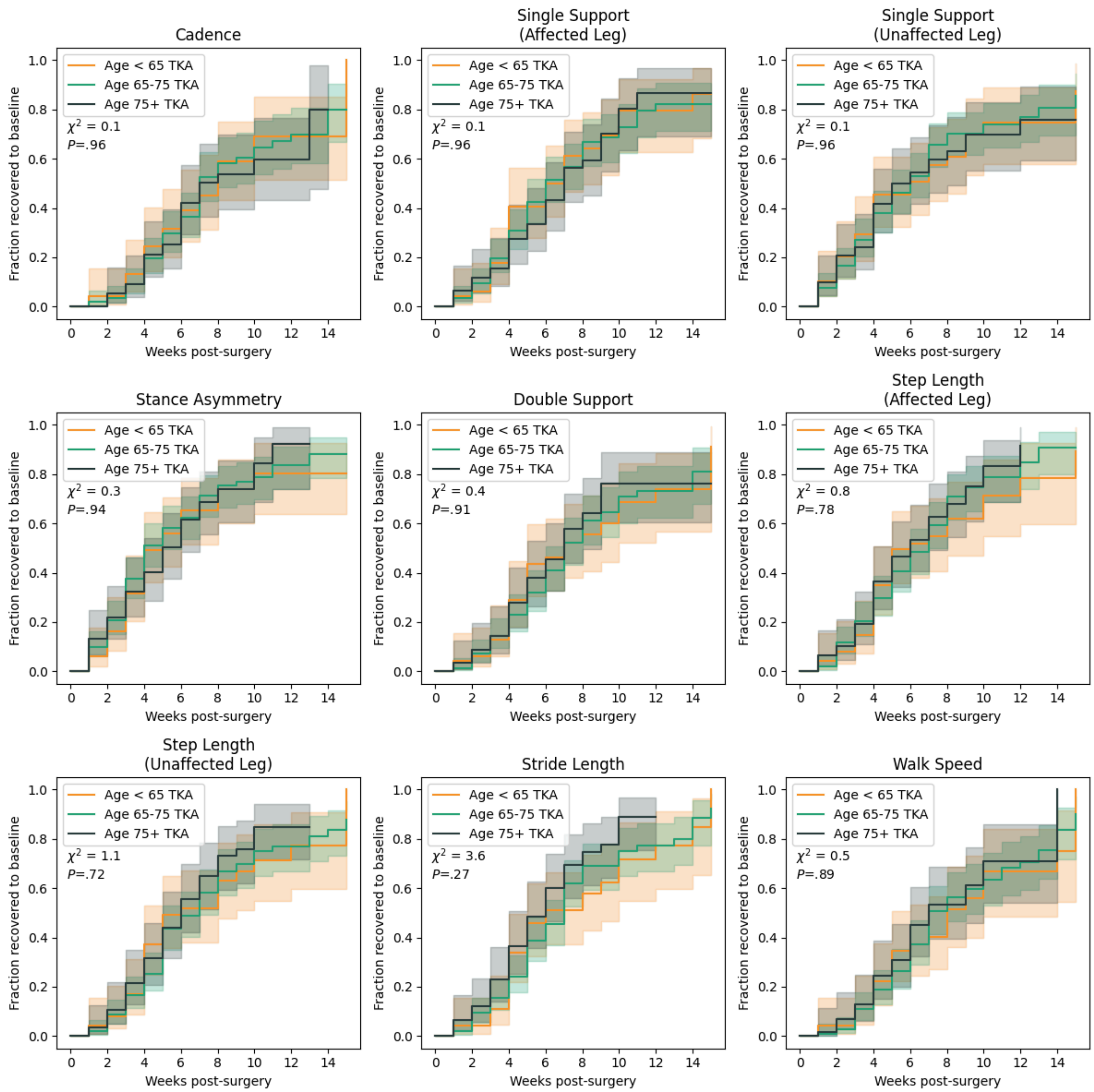

Figure S13 (continued).

a

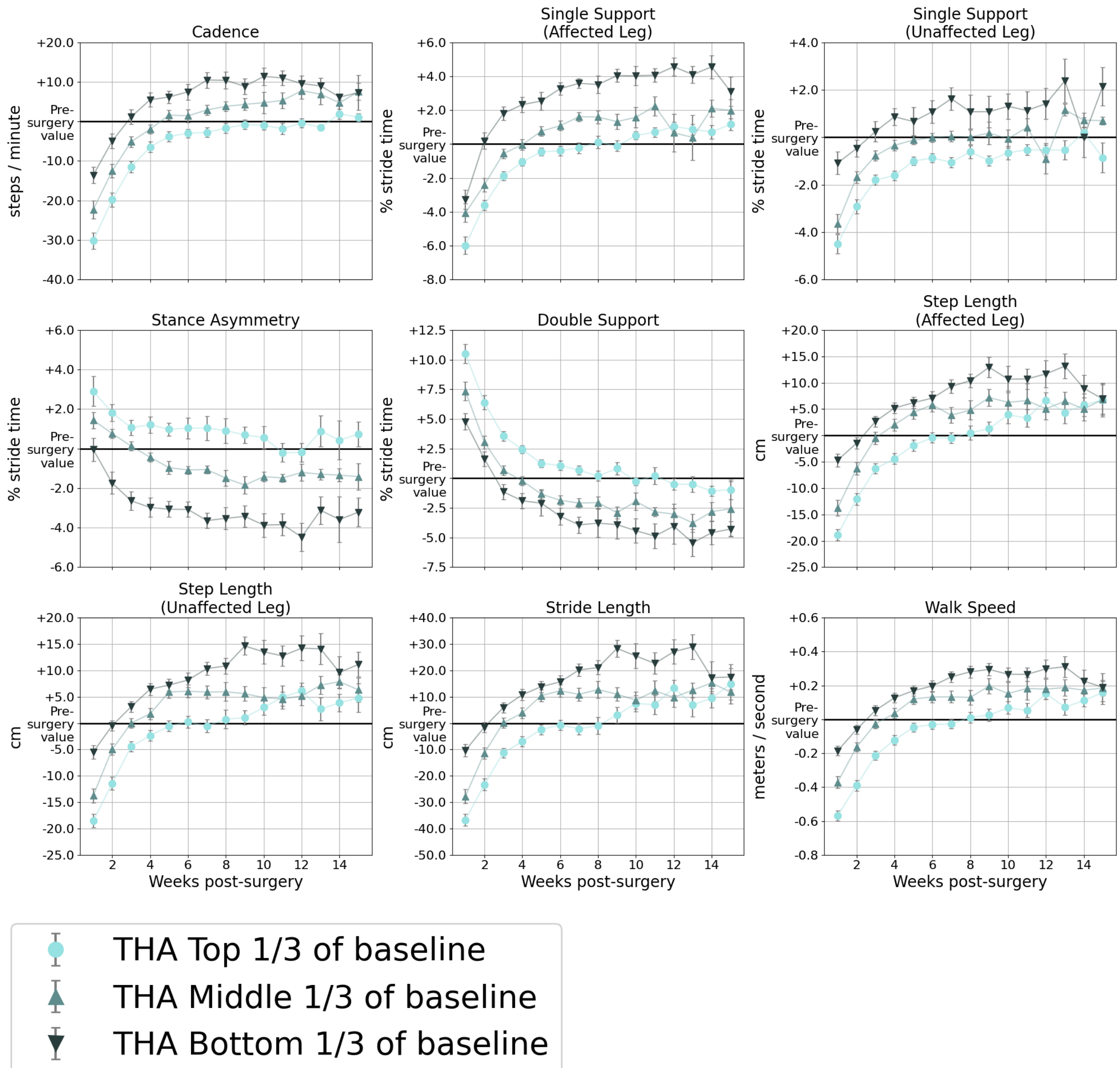

Figure S14. Recovery stratified by pre-surgery baseline level. a. THA in-app. **b.** TKA in-app. **c.** THA background. **d.** TKA background.

b

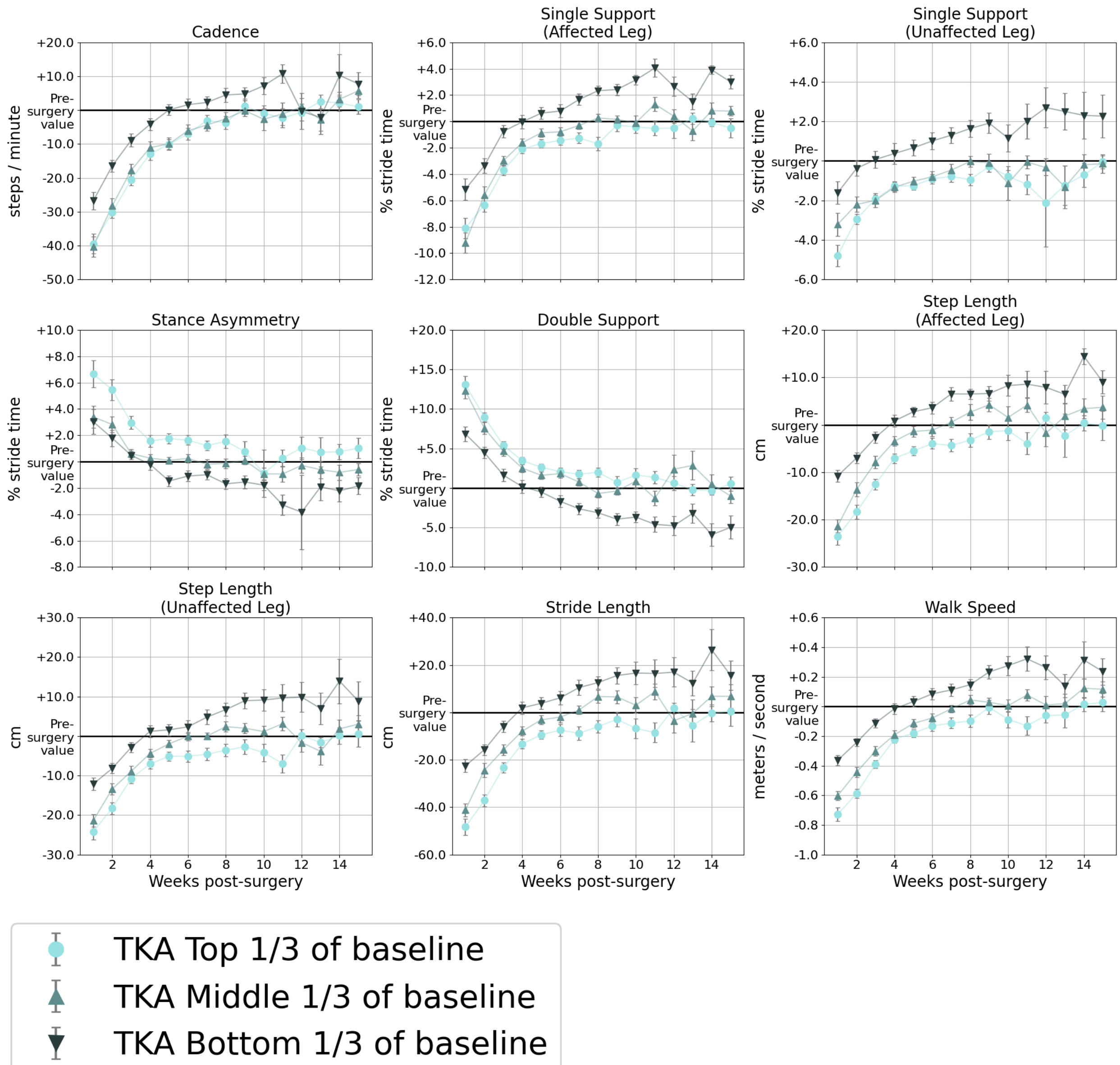

Figure S14 (continued).

c

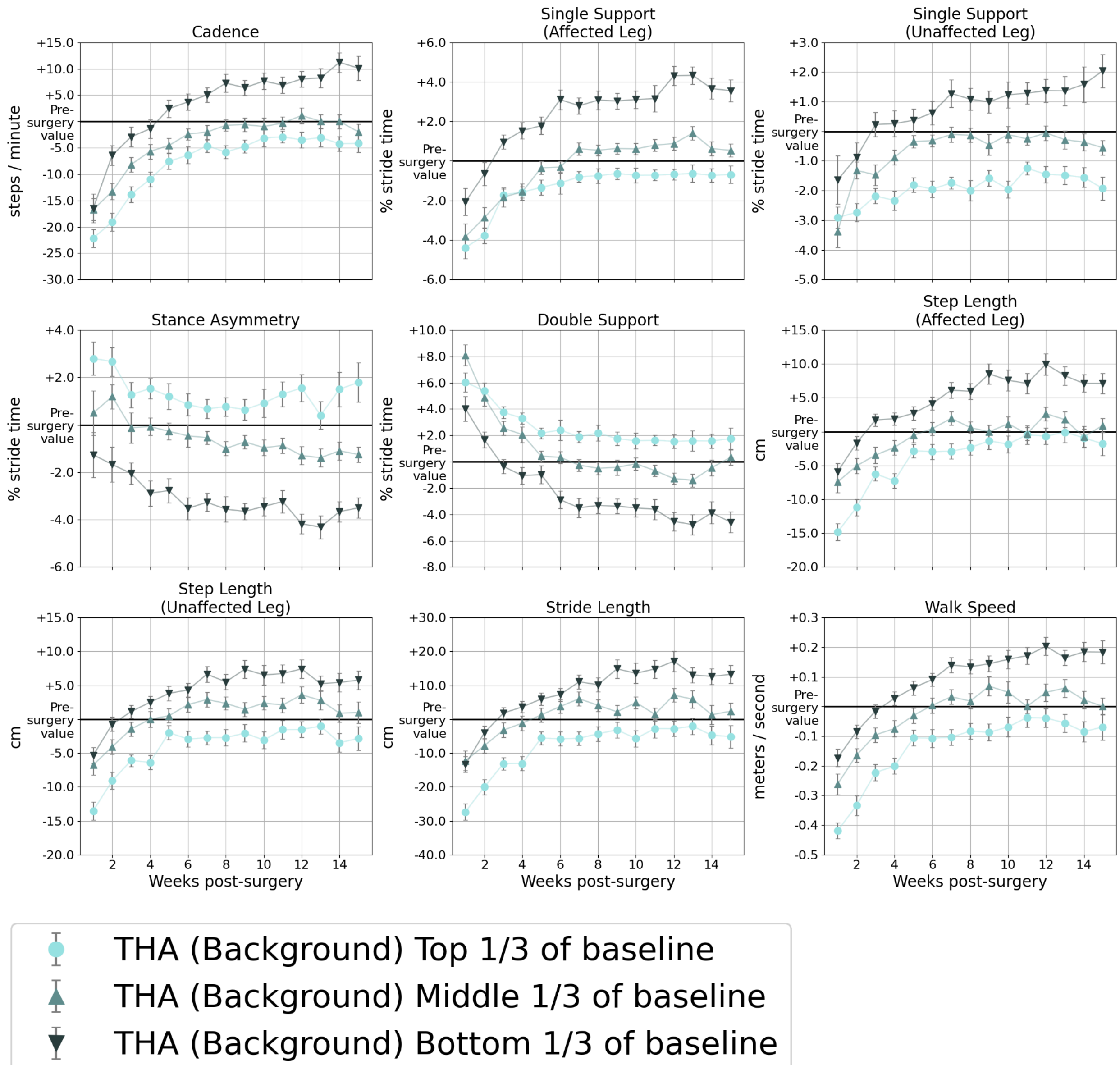

Figure S14 (continued).

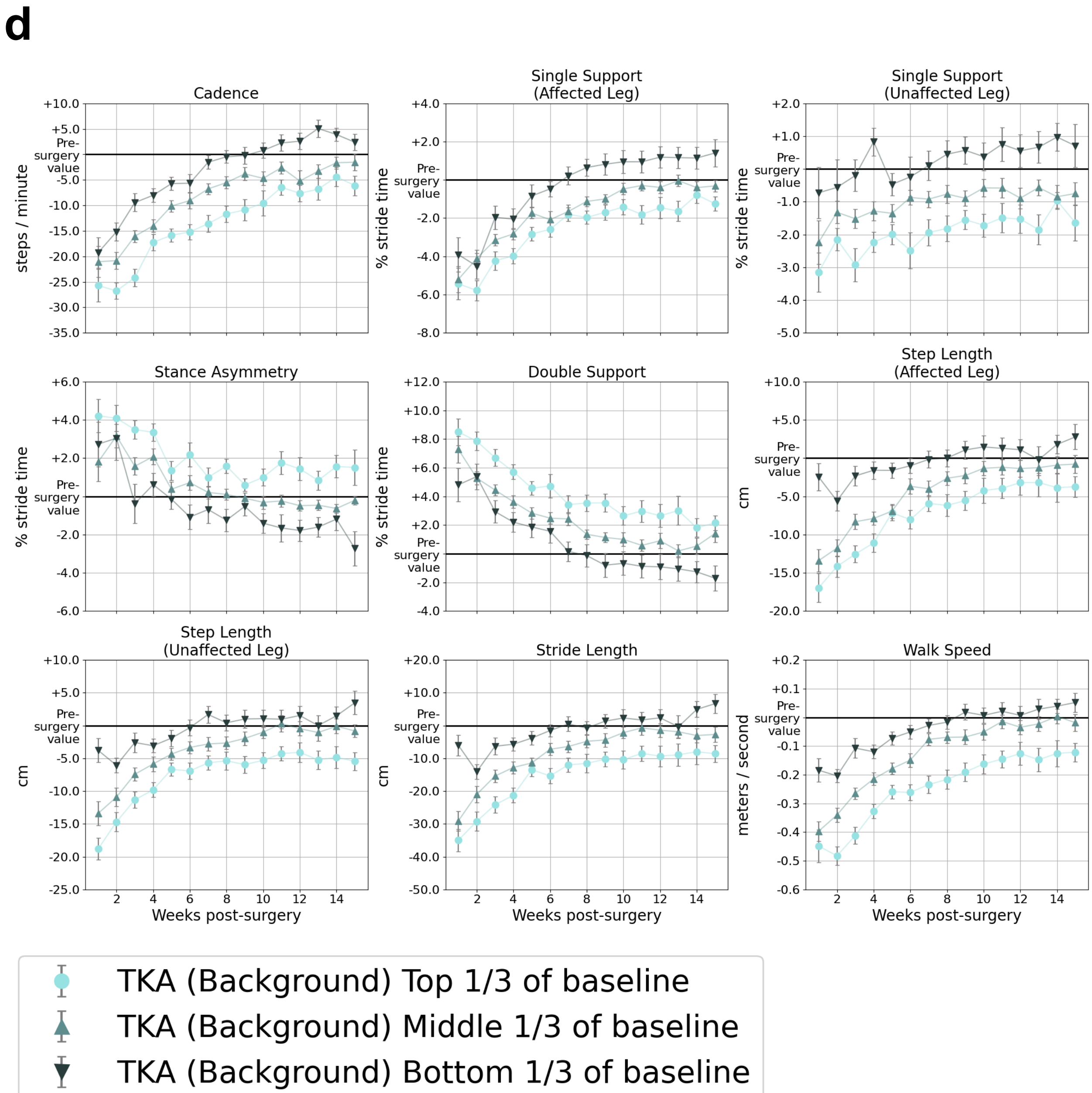

Figure S14 (continued).

a

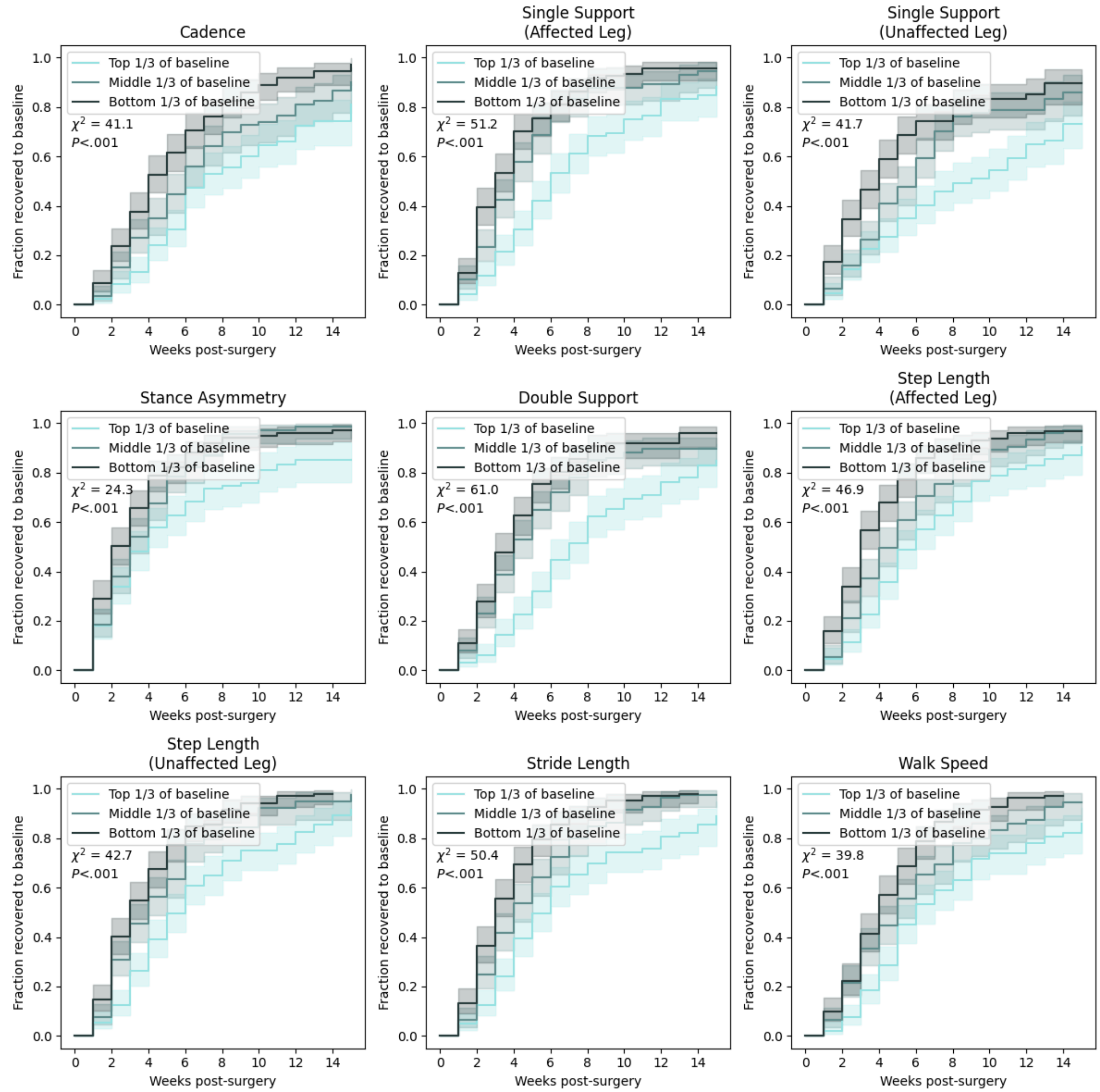

Figure S15. Kaplan-Meier analysis stratified by pre-surgery baseline. The curves show the fraction of patients whose average parameter value crossed their own pre-surgery gait level by each postoperative week. Shaded bands indicate 95% CIs. Each plot shows the χ^2 test statistic (df=2) and FDR-corrected P value of a multivariate log-rank test to determine if the curves differ significantly. **a.** THA in-app. **b.** TKA in-app. **c.** THA background. **d.** TKA background.

b

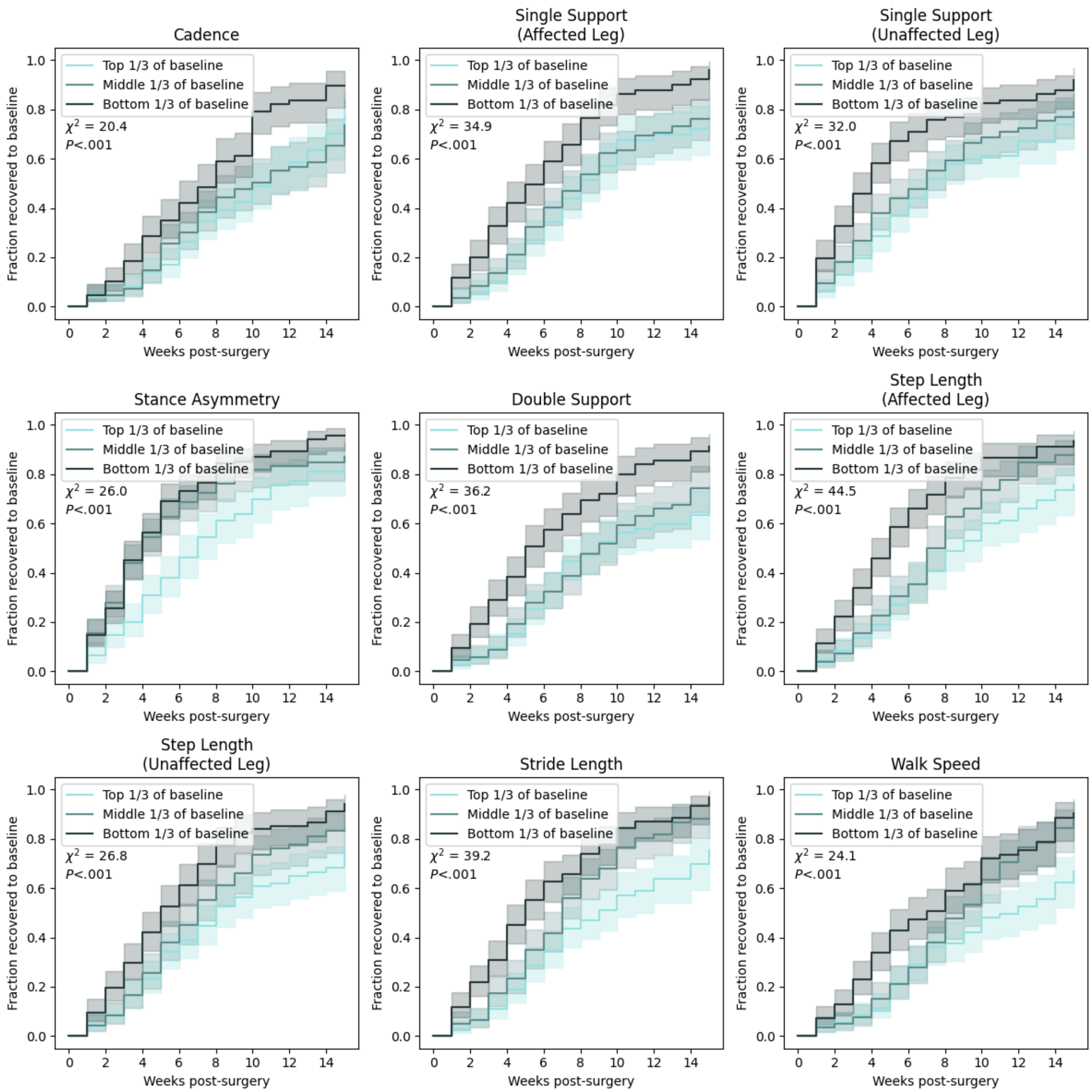

Figure S15 (continued).

c

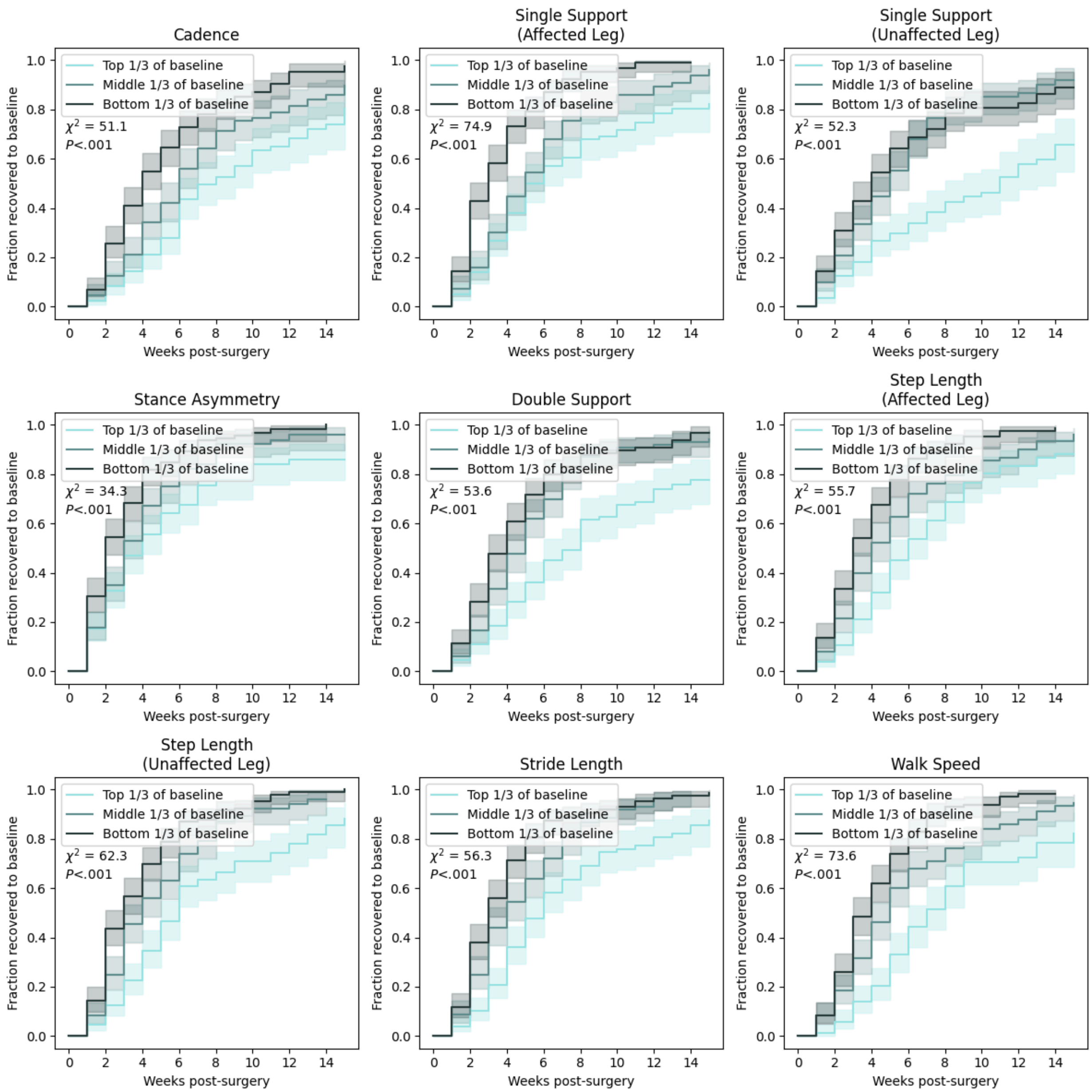

Figure S15 (continued).

d

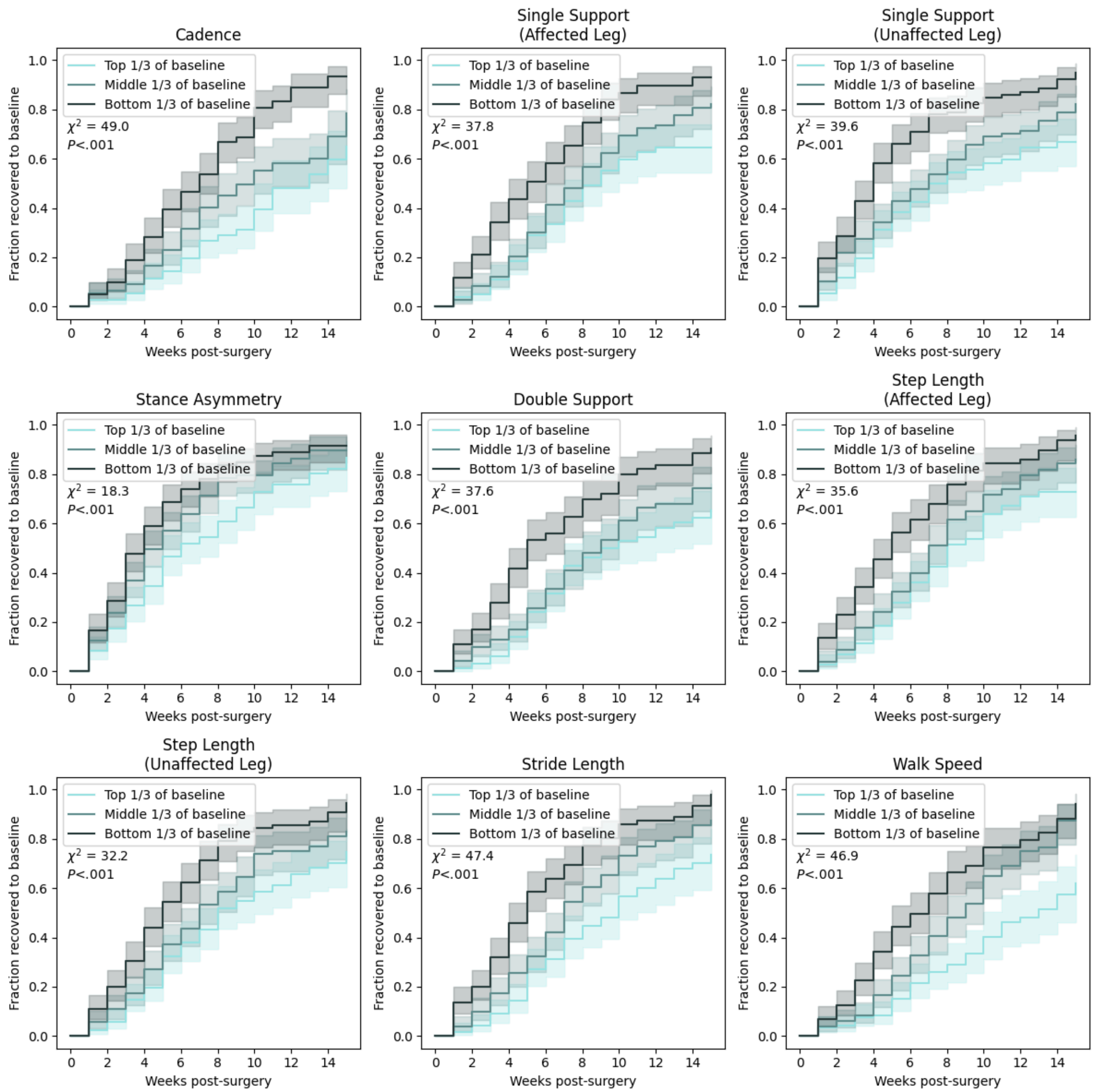

Figure S15 (continued).

a

b

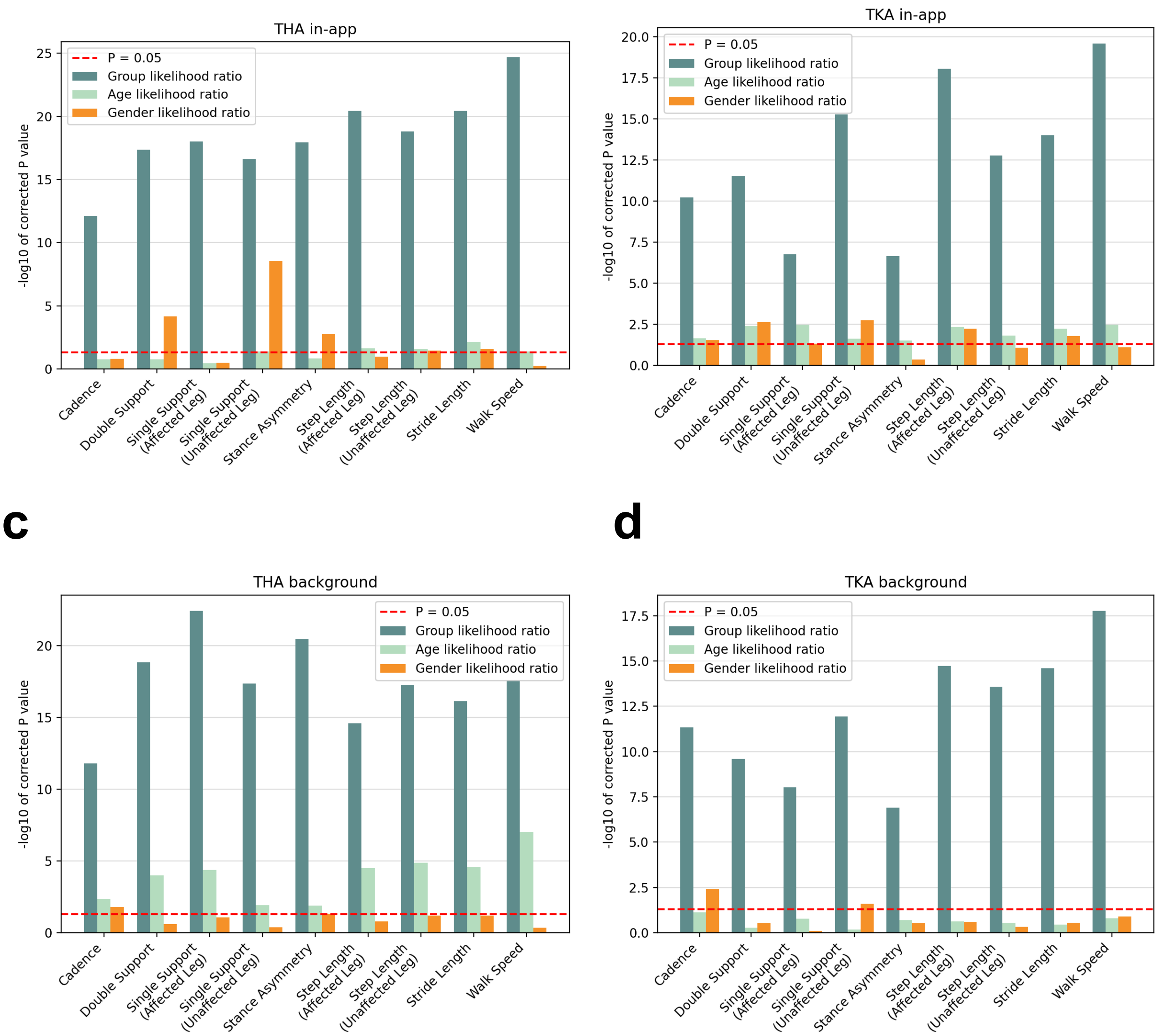

c

d

Figure S16. Likelihood ratio test (LRT) P values from nested linear mixed-effects models of pre-surgery baseline level. Models evaluated the contribution of key fixed effects across nine gait parameters. For each gait parameter, a full model including group (top vs. middle vs. bottom ⅓ of baseline), age, and gender (and the shared time/recovery-curve specification and random-effects structure) was fit by maximum likelihood. Three reduced models were then fit, each omitting one term (condition, age, or gender) while keeping all other terms and the random-effects structure identical. The plotted P values correspond to the LRT comparing each reduced model to its matched full model, quantifying the evidence that the removed term improves model fit for each parameter. All P values were adjusted for multiple testing using the Benjamini-Hochberg FDR method. **a.** THA in-app. **b.** TKA in-app. **c.** THA background. **d.** TKA background.

a

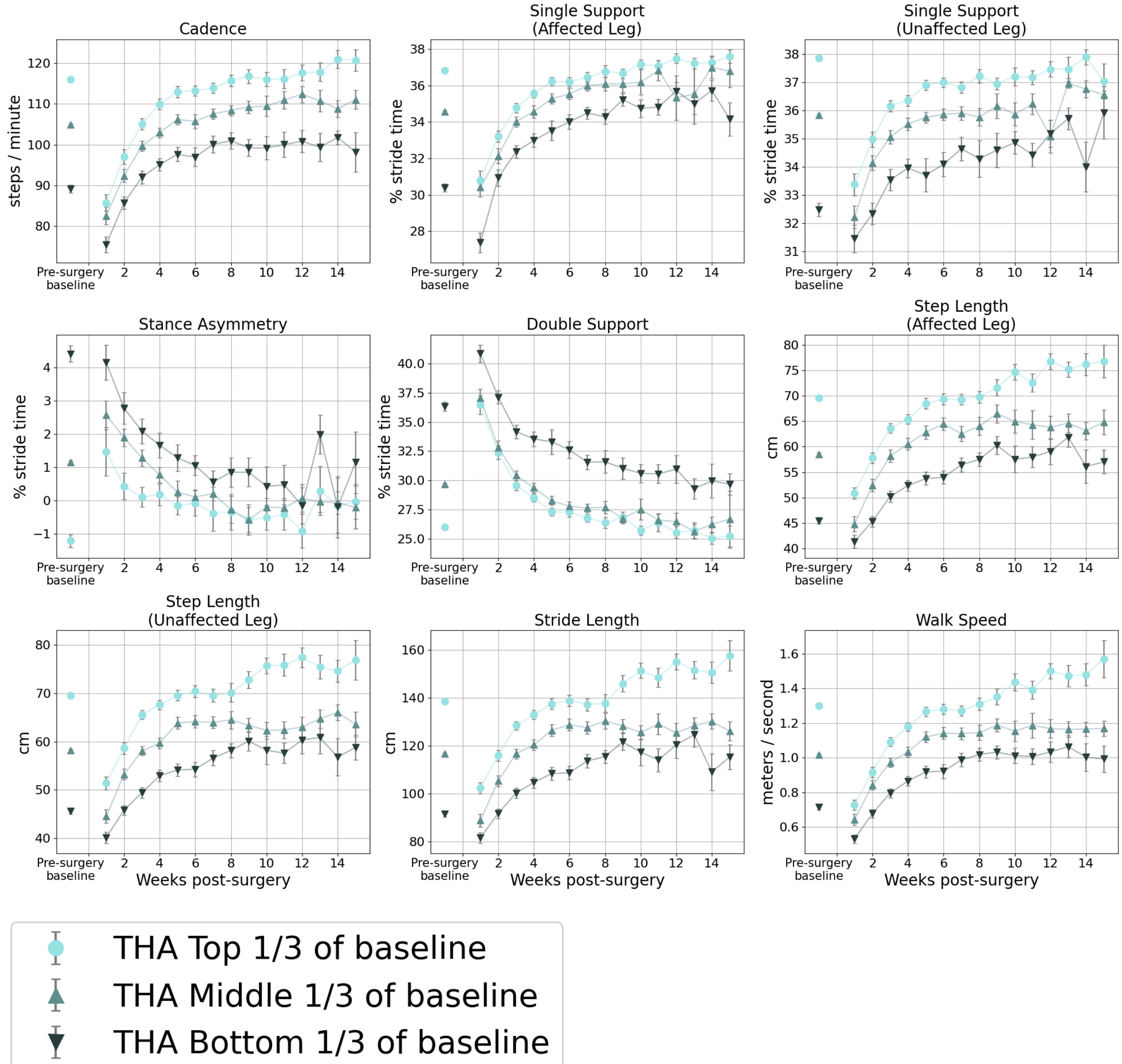

Figure S17. Non-normalized recovery stratified by pre-surgery baseline level. Raw parameter values at each week, not normalized by pre-surgery baseline values, are shown. Average pre-surgery baseline parameter values for each group are shown as the leftmost points on each graph, not connected with a line to the post-surgery recovery trajectory. **a.** THA in-app. **b.** TKA in-app. **c.** THA background. **d.** TKA background.

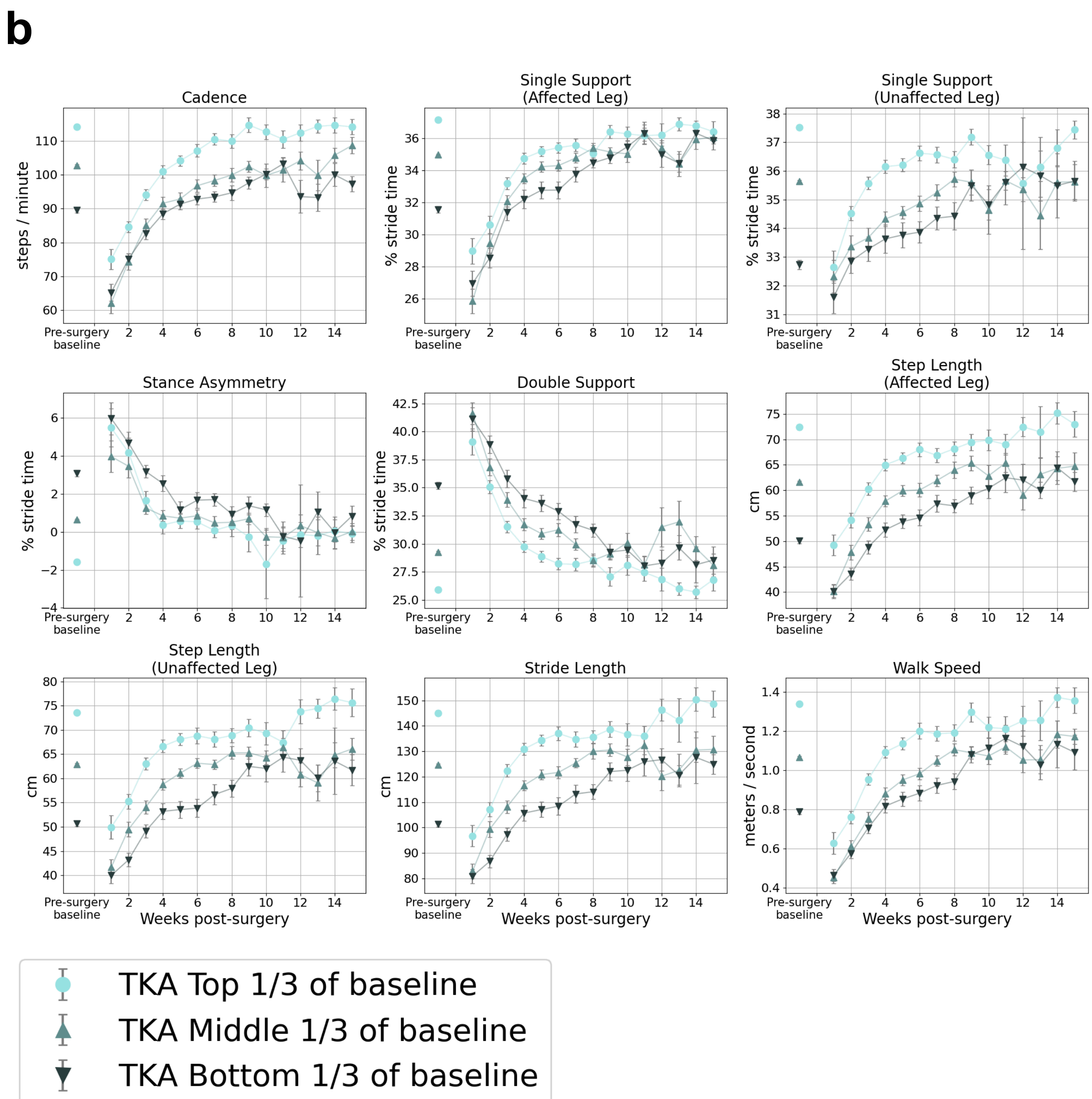

Figure S17 (continued).

c

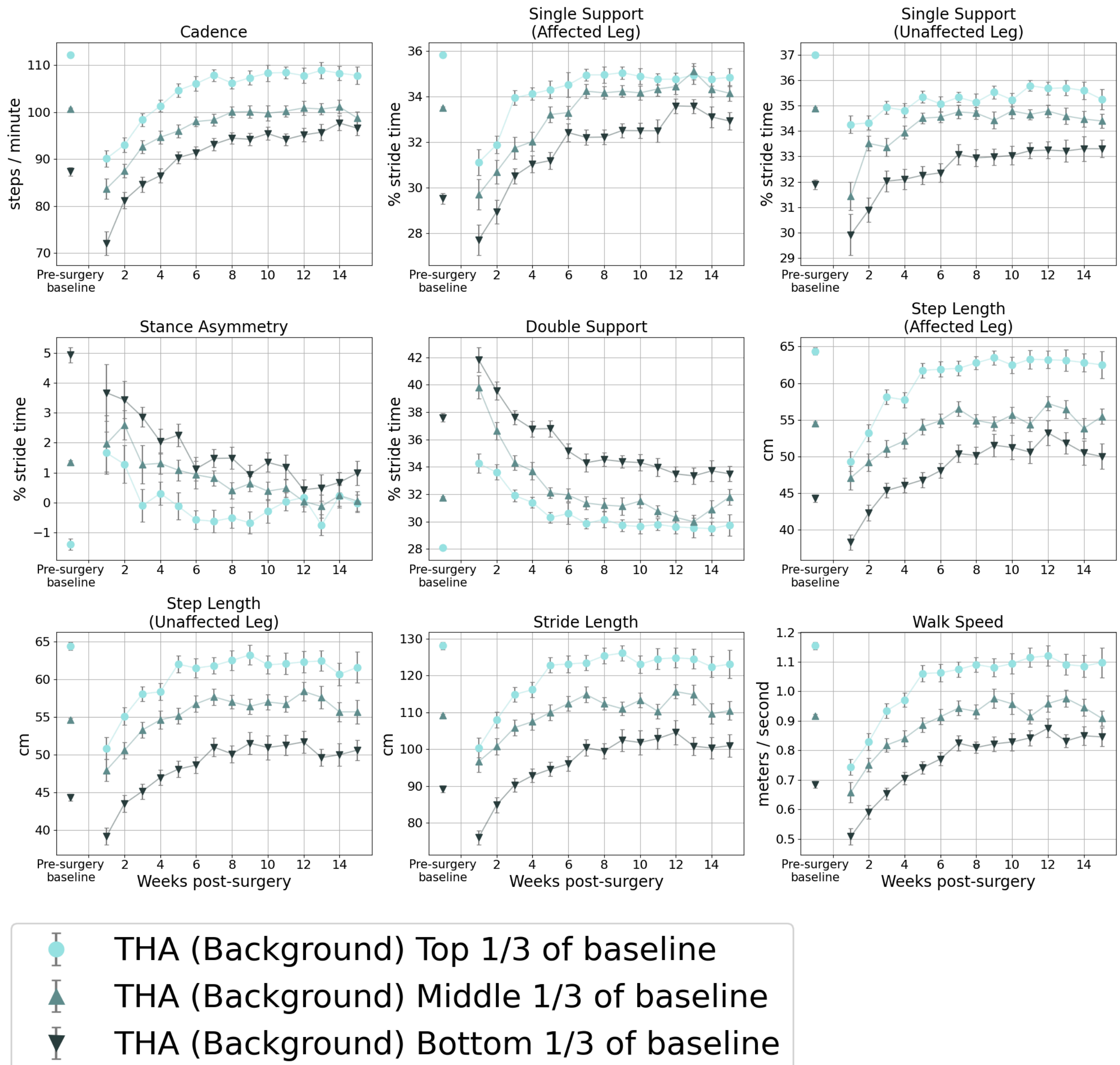

Figure S17 (continued).

d

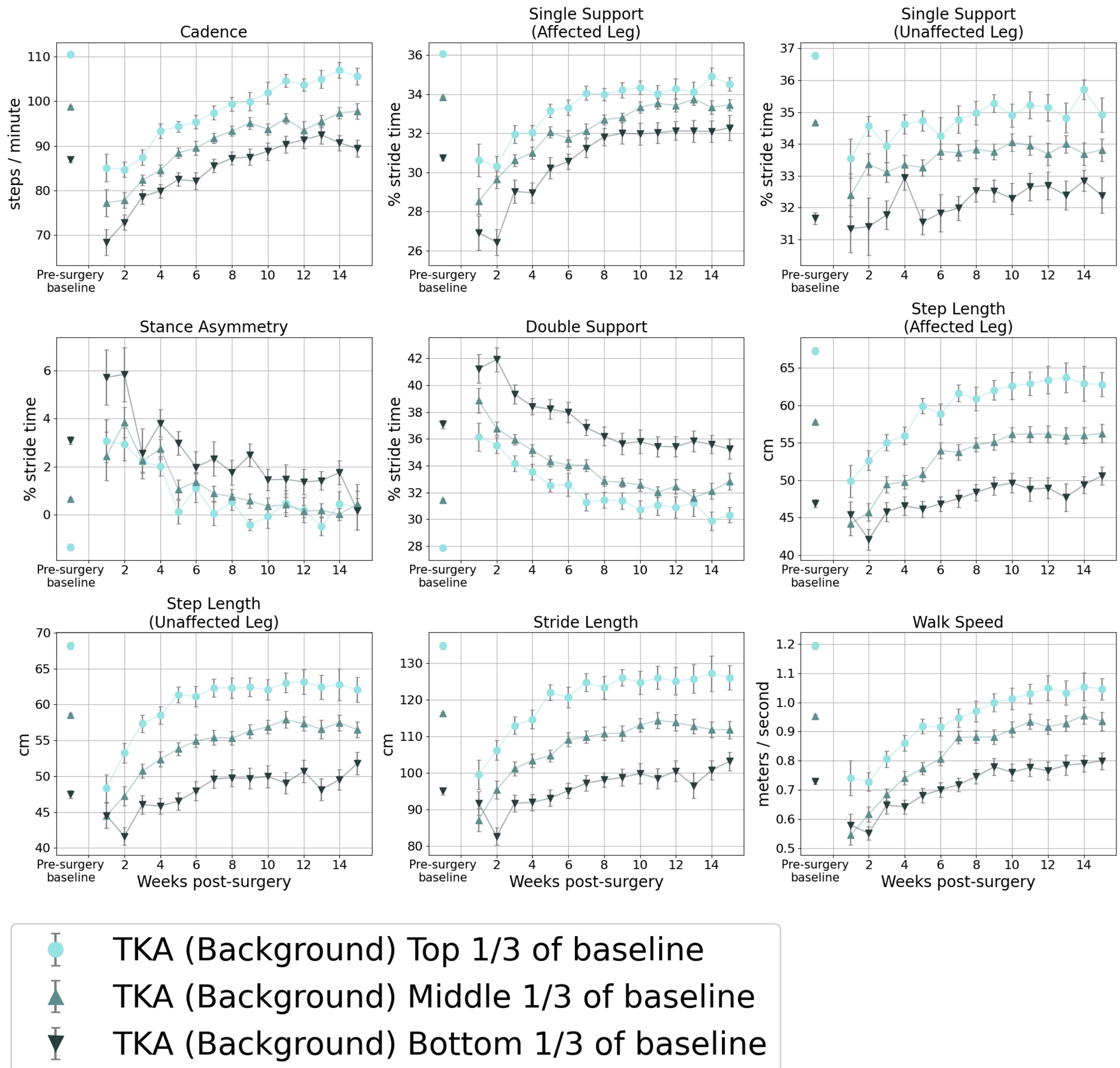

Figure S17 (continued).

Group	Parameter	Difference in EMM Between Groups (95% CI)	*z* score	P value	Corrected P value
THA vs. TKA	Cadence	5.58 (2.54, 8.62)	3.60	<.001	<.001
THA vs. TKA	Single Support Affected	1.71 (0.90, 2.52)	4.12	<.001	<.001
THA vs. TKA	Single Support Unaffected	0.73 (0.02, 1.44)	2.01	.04	.06
THA vs. TKA	Stance Asymmetry	-0.99 (-2.00, 0.03)	-1.91	.06	.07
THA vs. TKA	Double Support	-2.42 (-3.56, -1.27)	-4.14	<.001	<.001
THA vs. TKA	Step Length Affected	5.19 (2.96, 7.42)	4.56	<.001	<.001
THA vs. TKA	Step Length Unaffected	5.84 (3.57, 8.11)	5.04	<.001	<.001
THA vs. TKA	Stride Length	10.84 (6.56, 15.12)	4.96	<.001	<.001
THA vs. TKA	Walk Speed	0.14 (0.09, 0.20)	5.16	<.001	<.001
THA in-app vs. background	Cadence	4.68 (2.66, 6.71)	4.54	<.001	<.001
THA in-app vs. background	Single Support Affected	0.77 (0.21, 1.32)	2.72	.007	.01
THA in-app vs. background	Single Support Unaffected	0.40 (-0.11, 0.91)	1.55	.12	.14
THA in-app vs. background	Stance Asymmetry	-0.35 (-1.08, 0.37)	-0.96	.34	.35
THA in-app vs. background	Double Support	-1.16 (-1.94, -0.38)	-2.92	.003	.006
THA in-app vs. background	Step Length Affected	4.33 (2.79, 5.87)	5.52	<.001	<.001
THA in-app vs. background	Step Length Unaffected	4.89 (3.31, 6.46)	6.08	<.001	<.001
THA in-app vs. background	Stride Length	9.14 (6.20, 12.09)	6.09	<.001	<.001
THA in-app vs. background	Walk Speed	0.14 (0.10, 0.17)	7.51	<.001	<.001
TKA in-app vs. background	Cadence	2.43 (0.32, 4.54)	2.26	.02	.04
TKA in-app vs. background	Single Support Affected	0.51 (-0.07, 1.08)	1.73	.08	.1
TKA in-app vs. background	Single Support Unaffected	0.01 (-0.52, 0.54)	0.04	.97	.97
TKA in-app vs. background	Stance Asymmetry	-0.49 (-1.25, 0.26)	-1.28	.2	.22
TKA in-app vs. background	Double Support	-0.52 (-1.33, 0.29)	-1.26	.21	.22
TKA in-app vs. background	Step Length Affected	2.78 (1.17, 4.38)	3.39	<.001	.001
TKA in-app vs. background	Step Length Unaffected	1.85 (0.21, 3.49)	2.21	.03	.04
TKA in-app vs. background	Stride Length	4.59 (1.52, 7.67)	2.93	.003	.006
TKA in-app vs. background	Walk Speed	0.07 (0.04, 0.11)	3.92	<.001	<.001

Table S1. Comparison of late-recovery plateau levels. Paramter changes relative to baseline in weeks 11-15 post-surgery were calculated using the estimated marginal means (EMMs) of weeks 11-15 from the mixed-effects models used for the analyses described elsewhere. Difference in EMM between groups shows the difference in EMM at weeks 11-15 between the first group and the second group in the comparison. Units are the native units of the given parameter. (For example, the first row of the table shows that the EMM of the THA in-app group minus the EMM of the TKA in-app group is equal to 5.58 steps/minute.) For the Wald test used to measure significance of this difference, the *z* score test statistic and P value are reported. Corrected P values were adjusted for multiple testing using the Benjamini-Hochberg FDR method.

	THA In-app	THA BG	TKA In-app	TKA BG	Male THA In-app	Male THA BG	Male TKA In-app	Male TKA BG	Female THA In-app	Female THA BG	Female TKA In-app	Female TKA BG
Week 1	155	143	104	119	65	65	41	50	90	78	63	69
Week 2	185	181	140	152	78	90	53	66	107	91	87	86
Week 3	177	187	145	175	76	91	62	77	101	96	83	98
Week 4	169	181	145	178	71	84	59	81	98	97	86	97
Week 5	146	175	147	181	68	83	53	81	78	92	94	100
Week 6	135	167	128	162	63	78	47	75	72	89	81	87
Week 7	114	160	123	151	53	77	46	64	61	83	77	87
Week 8	91	146	97	144	42	69	35	65	49	77	62	79
Week 9	70	133	69	131	33	60	32	58	37	73	37	73
Week 10	51	113	56	109	25	52	23	51	26	61	33	58
Week 11	42	102	39	106	24	46	18	49	18	56	21	57
Week 12	48	102	29	100	26	45	11	44	22	57	18	56
Week 13	33	95	26	92	16	43	10	41	17	52	16	51
Week 14	29	74	27	87	17	35	11	43	12	39	16	44
Week 15	24	70	21	82	10	30	7	39	14	40	14	43

	THA In-app Age < 65	THA In-app Age 65-75	THA In-app Age 75+	THA BG Age < 65	THA BG Age 65-75	THA BG Age 75+	TKA In-app Age < 65	TKA In-app Age 65-75	TKA In-app Age 75+	TKA BG Age < 65	TKA BG Age 65-75	TKA BG Age 75+
Week 1	42	77	36	37	73	33	24	57	23	28	69	22
Week 2	49	88	48	53	90	38	27	84	29	33	90	29
Week 3	48	85	44	52	88	47	28	85	32	39	101	35
Week 4	43	80	46	51	86	44	35	78	32	45	95	38
Week 5	37	73	36	45	86	44	28	86	33	40	100	41
Week 6	34	62	39	45	76	46	27	74	27	35	92	35
Week 7	26	55	33	42	78	40	20	73	30	32	87	32
Week 8	18	44	29	36	75	35	18	57	22	29	82	33
Week 9	15	34	21	35	63	35	14	39	16	27	75	29
Week 10	12	25	14	33	56	24	11	32	13	21	60	28
Week 11	12	18	12	29	49	24	6	22	11	22	57	27
Week 12	12	24	12	27	51	24	7	13	9	22	53	25
Week 13	7	19	7	25	48	22	4	15	7	18	51	23
Week 14	6	15	8	19	39	16	8	15	4	20	46	21
Week 15	6	13	5	19	36	15	3	12	6	18	44	20

Table S2. Week-by-week patient counts. BG: background.

	Walk speed			Double support		
	Top third	Middle third	Bottom third	Top third	Middle third	Bottom third
Week 1	65	47	43	60	50	45
Week 2	74	60	51	68	64	53
Week 3	72	59	46	69	62	46
Week 4	64	54	51	60	62	47
Week 5	56	48	42	56	50	40
Week 6	46	50	39	48	51	36
Week 7	44	36	34	43	39	32
Week 8	30	32	29	35	30	26
Week 9	27	25	18	29	21	20
Week 10	21	15	15	24	10	17
Week 11	18	11	13	17	12	13
Week 12	20	17	11	22	13	13
Week 13	11	15	7	14	7	12
Week 14	14	8	7	17	5	7
Week 15	7	11	6	10	5	9

Table S3. Week-by-week patient counts of THA in-app walks split by patient baseline.
